# Oxidative stress-induced impairment of trophoblast function causes preeclampsia through the unfolded protein response pathway

**DOI:** 10.1038/s41598-021-97799-y

**Published:** 2021-09-16

**Authors:** Indrani Mukherjee, Ruby Dhar, Sunil Singh, Jai Bhagwan Sharma, Tapas Chandra Nag, Asit Ranjan Mridha, Parul Jaiswal, Subhrajit Biswas, Subhradip Karmakar

**Affiliations:** 1 Department of Biochemistry, All India Institute of Medical Sciences, New Delhi, India; 2 Amity Institute of Biotechnology (AIB), Amity University, Noida, India; 3 Department of Obstetrics and Gynaecology, All India Institute of Medical Sciences, New Delhi, India; 4 Department of Anatomy, All India Institute of Medical Sciences, New Delhi, India; 5 Department of Pathology, All India Institute of Medical Sciences, New Delhi, India; 6 Research Laboratory 101, J3 Block, Amity Institute of Molecular Medicine and Stem Cell Research (AIMMSCR), Amity University, Amity University Campus, Sector 125, Noida, Uttar Pradesh 201313 India

**Keywords:** Reproductive biology, Reproductive disorders, Molecular medicine

## Abstract

Pre-eclampsia (PE) is a pregnancy-specific disorder, characterized by hypertension and proteinuria. In PE, trophoblasts mediated inadequate remodeling of uterine spiral arteries seem to interrupt uteroplacental blood flow, one of the hallmarks in the early onset of PE (EO-PE). This, in turn, results in placental ischemia–reperfusion injury during hypoxia and reoxygenation episodes, leading to the generation of reactive oxygen species (ROS) and oxidative stress (OS). But still it is debatable if OS is a cause or consequence of PE. In this present study, we have investigated the effects of OS on PE placentae and trophoblast cell functions using BeWo and HTR8/SVneo cell lines. PE placental tissues showed abnormal ultrastructure, high level of reactive oxygen species (ROS) with altered unfolded protein responses (UPR) in compare with term placental tissues. Similar to PE placentae, during OS induction, the trophoblast cells showed altered invasion and migration properties with significantly variable expression of differentiation and invasion markers, e.g., syncytin and MMPs. The effect was rescued by antioxidant, *N*-acetyl cysteine, thereby implying a ROS-specific effect and in the trophoblast cells, OS triggers UPR pathway through IRE1α-XBP1 axis. Taken together, these findings highlight the harmful effect of unfolded protein response, which was induced due to OS on trophoblast cells and deformed invasion and differentiation programme and can be extended further to clinical settings to identify clinically approved antioxidants during pregnancy as a therapeutic measure to reduce the onset of PE.

## Introduction

Preeclampsia (PE), a common gestational complication, is a multifactorial crisis syndrome in pregnant females characterized by de novo onset of hypertension after 20 weeks of gestation. The etiology of PE is still enigmatic. Multiple factors are known to contribute to this condition, such as poor placentation, improper trophoblast invasion leading to endothelial dysfunction, oxidative stress, altered local and systemic immune regulation^1^. It is defined as early-onset hypertension with associated maternal organ dysfunction and/or fetal growth restriction complicating 5–8% of pregnancies worldwide, making it a leading cause of maternal and fetal morbidity and mortality^2^. Unlike normal pregnancy, where a subpopulation of fetal cytotrophoblast stem cells differentiate and invade the uterus and its arterioles, the cytotrophoblast cell differentiation is abnormal in PE^3^.

The pathophysiology of PE initiates in early pregnancy when there are deficient maternal spiral artery remodeling and insufficient placental perfusion due to inadequate or defective placentation^4^. This gives rise to placental ischemic reperfusion injury resulting in oxidative stress and generation of reactive oxygen species, thereby contributing to the clinical manifestations of PE^5^. Oxidative stress can pose various threats to cells, such as DNA damage, cell cycle arrest, senescence, oncogenic transformation and proliferation^6–9^. In context to the human placenta, adverse effects of oxidative stress have been observed, such as, aberrant chorionic villi formations, and placental function^10–12^. This current study is aimed to understand the role of OS in the molecular pathogenesis of PE.

In the human placenta, villi formation initially starts over the entire surface of the chorionic sac and, towards the beginning of the first trimester, this villi over the superficial pole regress to leave the definitive discoid placenta^3,5^. This physiological process is considered to be ROS driven^13^. Pathophysiological mechanisms affected and responsible for PE are still obscure, but it is known that imbalance between the generation of oxidative stress and antioxidant defense system is associated with altered placental growth during the early phase of pregnancy^10,11,13^. Oxidative stress is generally characterized by the formation of large amounts of reactive oxygen species (ROS) in cell membranes, endoplasmic reticulum, and mitochondria^6^. ROS generated within the cells further contributes to mitochondrial structural damage. This process often leads to a vicious cycle generating more ROS due to an electron transport chain ^6–8^. However, the association between ROS and endoplasmic reticulum (ER) stress can also be considered as a major driving factor because ER has been recognized as a major center for the coordination of a vast array of cellular responses^14^. ER stress response of the cell is essentially mediated by activation of the unfolded protein response (UPR) pathway^13^, prolonged activation of which may lead to apoptosis of trophoblasts during PE^15^. In homeostatic conditions, the UPR pathways are maintained in an inactive state due to glucose-regulated protein 78 (GRP78) binding to the three UPR-associated proteins: inositol-requiring enzyme 1α (IRE1α), protein kinase RNA-like endoplasmic reticulum kinase (PERK), and activating transcription factor 6 (ATF6)^16,17^. Studies have found the involvement of GRP78 in syncytialization where reduced expression of this protein in trophoblast cells alters their fusion and differentiation^18,19^. However, the importance and involvement of UPR response during trophoblast differentiation remain largely unexplored.

Oxidative stress can be accompanied by the enhanced inflammatory response, which can cause vascular endothelial damage, increased trophoblast apoptosis, cellular senescence, and hampered growth factor transport due to reduced blood flow, which together culminates into placental dysfunction^6–9^. Proper placentation is the outcome of trophoblast cells mediated adhesion, invasion, and spiral artery remodeling for adequate placental perfusion, with trophoblast differentiation^20,21^. Mono-nucleated cytotrophoblast (CTB) cells fuse to form multinucleated syncytiotrophoblast (STB) cells, which form the barrier between maternal and fetal parts^20–22^. These fused cell layers serve major functions such as hormone production, and mediating nutrient and gas exchange. The trophoblast fusion is highly regulated, and its defect can have severe effects on the feto-placental unit^20–22^. Villous CTB (vCTB) fusion has to be tightly regulated since a limited fusion rate may lead to an abnormal STB layer formation and placental function deficit. Expression of fusogenic proteins such as Syncytin-1, Syncytin-2, their receptors SLC1A5, and MFSD2A respectively are known to be required for this STB formation process^23–25^. Expression of these fusion-related genes is affected by several factors^26,27^. Altered expression of Syncytin has been associated with placental pathophysiology, including PE^28^. Apart from this, multiple mechanisms has been attributed with PE, such as inefficient and shallow trophoblast invasion as well as incomplete remodeling of maternal spiral arteries leading to an increased uteroplacental vascular resistance. Trophoblast cells emerging from the distal end of the CTB cell columns of the anchoring villi are mitotically quiescent. These cells can differentiate and acquire EMT-like features enabling them to migrate into the decidua basalis of the endometrium. These are called the extra-villous trophoblasts (EVT). Once into the maternal decidua, this population of migrating trophoblasts, now called the interstitial CTB (iCTB) can invade the endometrium due to the release of matrix-degrading enzymes. A lineage of the interstitial EVT (iEVT) called the endovascular CTB (eCTB) further invades the uterine arteries remodeling them to transform into low resistance vessels. Uterine arteries undergo a series of remarkable pregnancy-specific changes that involve the replacement of endothelium and smooth muscle cells by eCTB loss of vascular elasticity dilation of the uterine arteries^2,29^. Improper and inappropriate eCTB invasion during the first half of pregnancy is found to be associated with early-onset preeclampsia due to a reduced blood flow to the placenta.

Even though considerable information on the pathophysiology of PE is known, there is a shortage of knowledge regarding the role and mechanism of oxidative stress accompanied by ER stress in altering the differentiation and syncytialization process of trophoblast cells. Proper placentation is an outcome of a well-coordinated invasion and differentiation program mediated by trophoblast cells. Therefore, we aimed to understand how oxidative stress and ER stress can contribute in promoting PE involving the UPR pathway. The effect of oxidative stress on trophoblast cell invasion and differentiation was studied using two different cell lines HTR8/SVneo and BeWo. Oxidative stress and thus induced ER stress attenuated the invasion and differentiation behavior of trophoblast cells.

## Results

### Reduced expression of differentiation and invasion markers in placental tissue from PE patients

**Figure 1:**
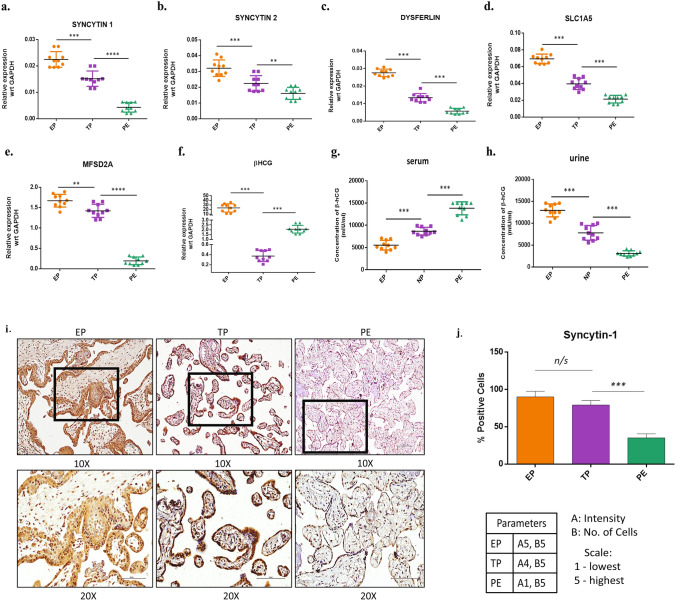
Expression of differentiation markers in PE tissues compare with normal subjects. **(a–f)** qPCR showing relative mRNA expression across the tissue samples i.e. EP (early placental villi, n = 10) TP (term placenta, n = 10), and PE (pre-eclampsia placenta, n = 10) respectively. The results were analyzed by the 2^−△CT^ method (△CT = CT value of sample −CT value of internal reference gene). **(g,h)** Comparative quantification of β-hCG in the serum and urine of PE patients with EP, and NP using ELISA assay. **(i)** Immunohistochemistry showing Syncytin-1 expression in the tissue sections from EP, TP, and PE placentas. **(j)** Relative quantification Syncytin-1 expression in the tissue sections from EP, TP, and PE placentas. All data are shown as mean ± standard deviation. Results are representative of at least ten independent experiments. *p < 0.05; **p < 0.01; ***p < 0.001.

One of the factors determining the outcome of a successful pregnancy is proper placentation. Two critical functions of the trophoblast cells, i.e., invasion and differentiation, are required for embryo implantation and to establish functional feto-maternal communication. The invasion is mediated by the regulated expression of proteases and protease inhibitors by trophoblast cells. While the expression of genes such as beta-human chorionic gonadotropin (β-hCG) and Syncytin is required for trophoblast differentiation in syncytiotrophoblast cells, which forms the placental barrier and establishes the nutrient circulation between the mother and fetus. To delineate the effect of ROS on the trophoblast differentiation process, we performed RT-PCR based quantification of the differentiation markers (SYN1, SYN2, DYS, SLC1A5, MFSD2A,) in placental tissues obtained post-delivery from both normal as well as PE patients (Suppl. Figure 1). Results showed a reduced expression of these differentiation markers in PE than the normal subjects (Fig. 1a–e). On the contrary, hCG expression was found to be elevated in serums from PE (Fig. 1g) as per reported in other finding^30^. Urinary hCG, however, showed the opposite trend as seen in the tissue (Fig. 1f,h), may be due to it’s retention by kidney^31^. Immunohistochemical analysis in placental tissues showed significantly reduced expression of Syncytin-1 in PE as compared to the normal term. (Fig. 1i,j). IgG control was shown to validate Syncytin-1 expression (Suppl. Figure 2).

**Figure 2:**
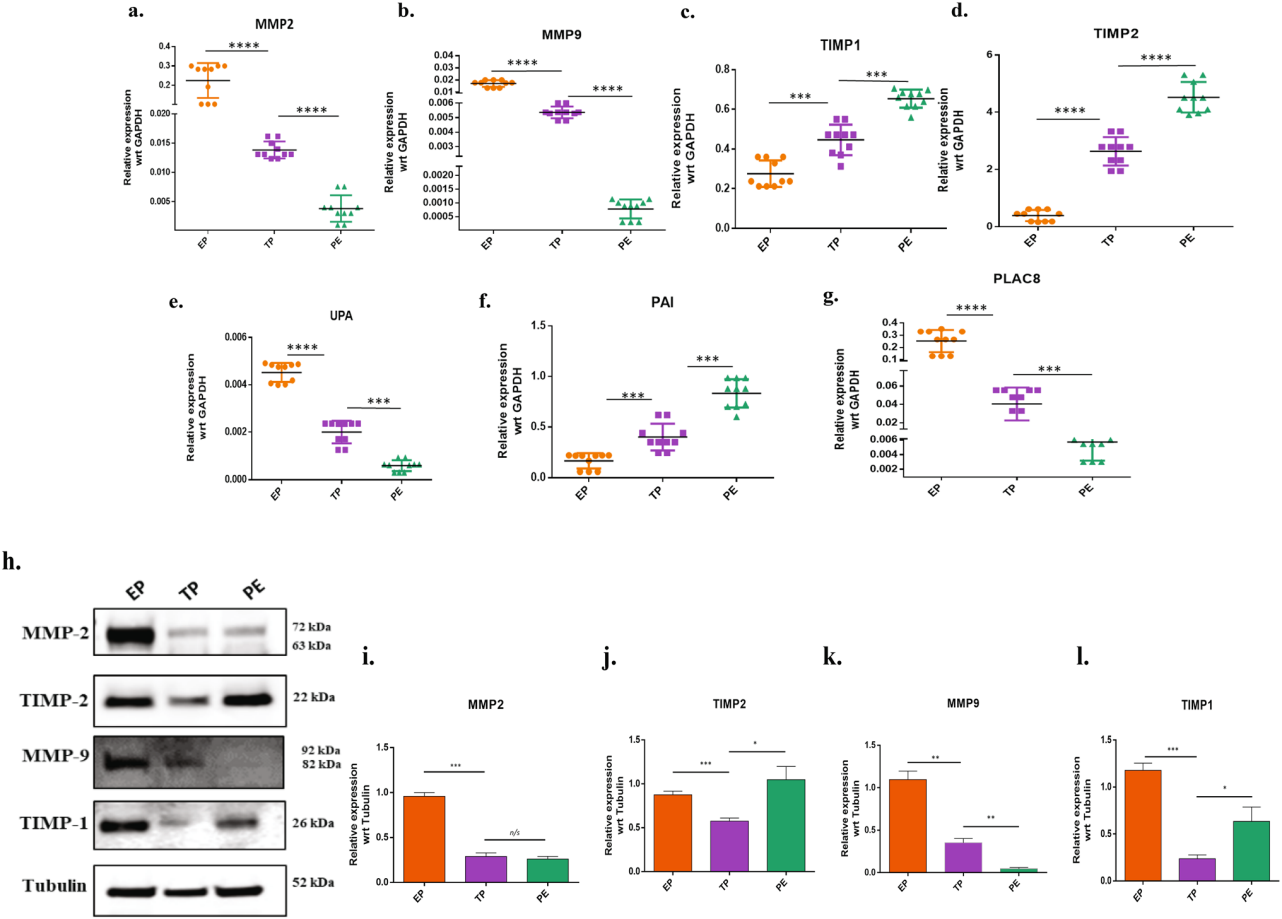
Expression of proteases and protease inhibitors in PE tissues. **(a–g)** qPCR showing relative mRNA expression for each of the genes MMP-2, MMP-9, TIMP-1, TIMP-2, UPA, PAI, and PLAC-8 in EP, TP, and PE tissues (n = 10). The results were analyzed by the 2^−△CT^ method (△CT = CT value of sample −CT value of internal reference gene). **(h)** Western blotting showing expression profile of proteases' protein and protease inhibitors. EP, TP, and PE tissue lysates were immunoblotted with MMP-2, MMP-9, TIMP-1, and TIMP-2 antibodies. α-Tubulin served as a loading control. **(i–l)** Protein expressions were quantified using ImageJ and plotted graphically after normalized with α-Tubulin values. All data are shown as mean ± standard deviation. Results are representative of ten independent experiments. *p < 0.05; **p < 0.01; ***p < 0.00; ****p < 0.0001.

Along with the differentiation markers, we also investigated the mRNA expression of the trophoblast proteases in PE. There was a significantly lower expression of the major proteases (MMP-2, MMP-9 & uPA) in PE as compared to term. Further, a significantly higher (p < 0.05) expression of protease inhibitors (TIMP-1, TIMP-2 & PAI-1) was also observed in PE (n = 10). As a comparison, early villi tissues showed the highest expression of MMP-2, MMP-9 & uPA. Interestingly, we observed ONZIN/PLAC8 (Placenta associated 8) expression to be significantly low in the PE (p < 0.001) tissues, as compared to term (Fig. 2a–g). PLAC8 was reported to be associated with invasive and migratory^24^. Western blotting was performed to confirm our RT-PCR results (Fig. 2h). Results showed significantly reduced expressions of MMP-9 (p < 0.001) in PE tissues compared to term placenta as seen earlier in PCR. However, there was no significant difference in the MMP-2 (72 kDa) protein expression (Fig. 2i,j). MMP inhibitors, TIMP-1, and TIMP-2 expression were also significantly higher (p < 0.01) in the PE as compared to the term (Fig. 2k,l). Overall, the expression of proteases was reduced while the expression of protease inhibitors was increased in PE tissues.

### Ultrastructural features alter in PE placenta

**Figure 3:**
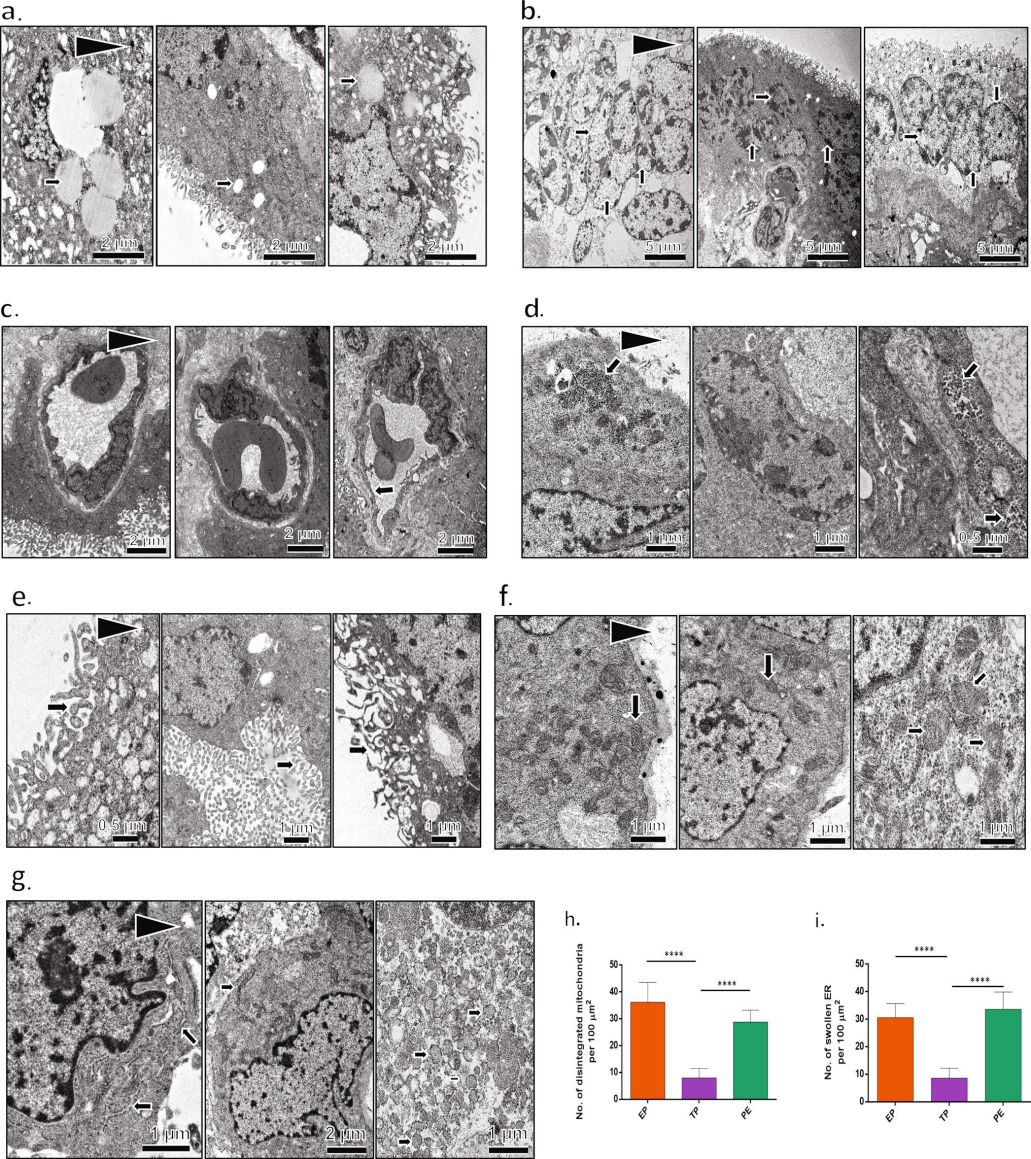
Ultrastructural changes in PE tissues in compare with healthy control. Transmission electron micrographs showing ultrastructual features among EP, TP, and PE tissue samples. Arrows were indicated for individual parameters in each image. **(a)** Lipid droplet profile. PE tissues have high lipid content. (**b)** Syncytiotrophoblasts. **(c)** Capillary endothelium. PE tissues showed thin and disrupted endothelium. **(d)** Glycogen content. PE tissues possessed a high amount of glycogen. **(e)** Microvilli. Microvilli were small, fragmented, and reduced in a number of PE tissues. **(f)** Mitochondrial profile. PE tissues have reduced the number of mitochondria and also compromised mitochondria. **(g)** PE tissues have distorted and swollen ER. **(h)** The number of distorted mitochondria was plotted graphically in EP, TP and PE tissues. **(i)** The number of swollen endoplasmic reticulum was plotted graphically in EP, TP and PE tissue samples. Results are representative of at least ten independent experiments. ****p < 0.0001.

We next investigated the cause for the altered trophoblast function in PE with speculation of ROS-mediated intracellular injury. Using TEM, we explored the ultrastructural details of placental tissues from PE and health controls. Significant ultrastructural changes were observed in the PE tissues (n = 10) compared with the healthy term placenta. Large lipid droplets (Fig. 3a), Syncytiotrophoblasts (Fig. 3b), thin endothelium (Fig. 3c), and high glycogen content (Fig. 3d) were observed in PE tissue. PE tissue sections had distorted, short microvilli (Fig. 3e), measuring 200–300 nm in length, while normal placental villi were 1 µM long. Interestingly, we observed bunches of disintegrated mitochondria (Fig. 3f) and swollen ER (Fig. 3g) in the PE tissues. We have quantified the number of disintegrated mitochondria between EP, TP and PE tissues (Fig. 3h.). There was a notable increase in the number of disintegrated mitochondria in PE tissues in compare with TP tissues. Similar histogram was prepared to show the numbers of the damaged mitochondria among EP, TP and PE tissues (Fig. 3i). PE tissues had significantly more number of swollen endoplasmic reticulum compared to TP tissues. The term tissues were rich in healthy mitochondria and had normal ER (Fig. 3f,g).

### PE placental tissues showed altered the unfolded protein responses with elevated ROS

**Figure 4:**
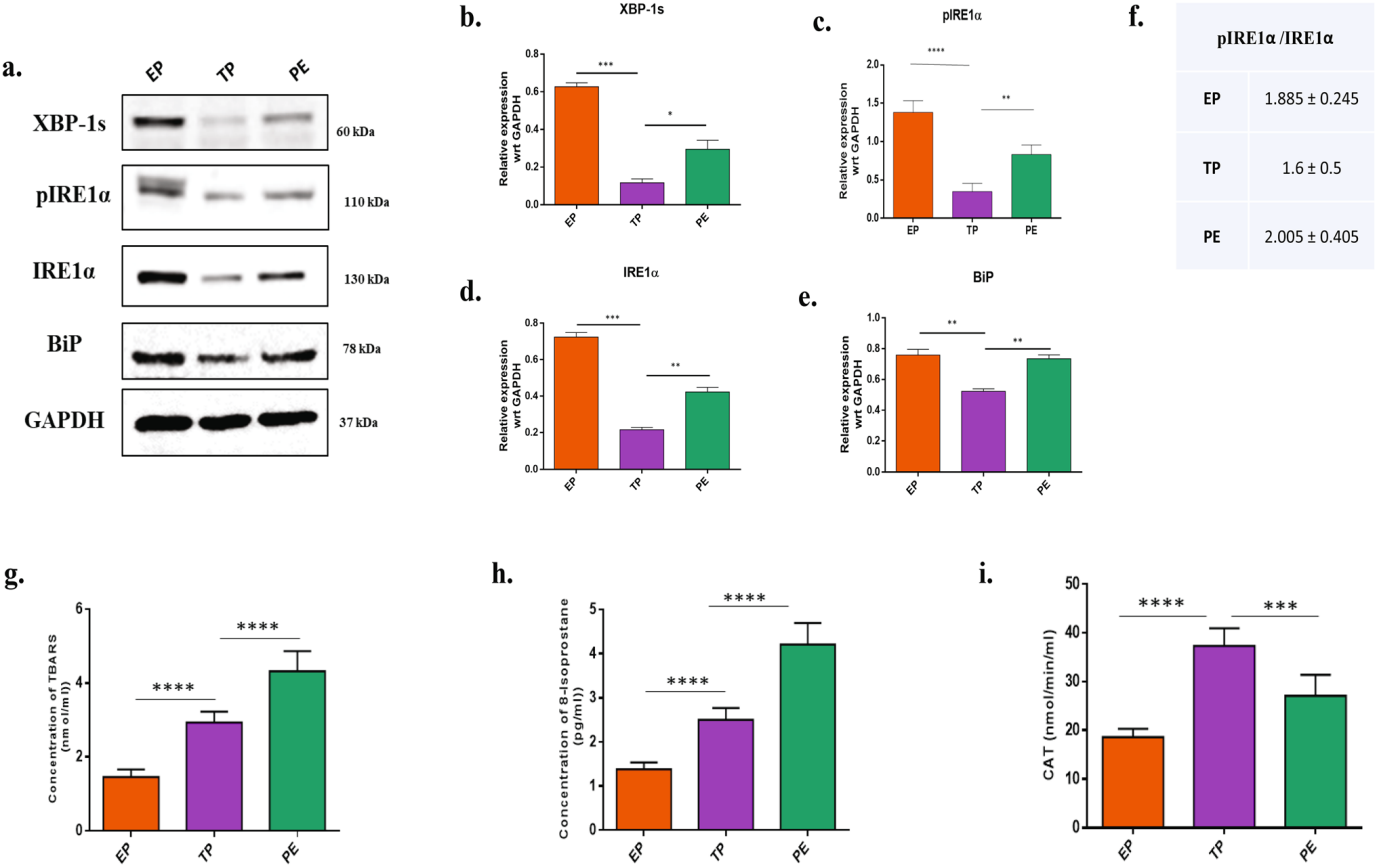
Altered UPR pathway with elevated ROS were associated with PE. (**a)** Western blots showing expression of proteins involved in UPR pathway. EP, TP, and PE (n = 10) lysates were immunoblotted for BiP, pIRE1α, IRE1α, XBP-1s. GAPDH served as a loading control. **(b–e)** Protein expressions were quantified using ImageJ and plotted graphically after normalized with GAPDH value. ROS levels were measured by quantifying the levels of oxidative stress markers. **(f)** Ratio of pIRE1α/IRE1α was calculated to confirm the activation of UPR pathway in EP, TP and PE tissue lysates, (**g**) Lipid peroxidation product TBARS, **(h)** 8-Isoprostane and **(i)** the antioxidant enzyme catalase activity were quantified in EP, TP, PE tissue lysates (n = 10) using ELISA, and data are plotted graphically. All data are shown as mean ± standard deviation. Results are representative of at least ten independent experiments. *p < 0.05; **p < 0.01; ***p < 0.001, ****p < 0.0001.

Structural changes of ER are usually an outcome of ER stress that often is associated with activation in the UPR pathway. To confirm our hypothesis, we performed western blotting for proteins associated with UPR. There was significant (p < 0.01) upregulation of BiP, pIRE1α, IRE1α and XBP-1s proteins in PE placental samples as compared to the normal term placenta (Fig. 4a–e). To confirm the activation of unfolded protein response pathway, the ratio of pIRE1α/IRE1α was done. Results showed that pIRE1α was significantly higher in the PE tissues compared to the TP tissues (Fig. 4f). In this context, the early villi displayed higher expressions of UPR indicating a differential activation of placental unfolded protein response pathways with heterogeneity between early villi and term placenta, which may be due to rapidly rising oxygen tension and adaptation of in the antioxidant defence^7^**.** Here, we investigated also the OS-mediated induction of ROS in these PE, EP, and TP placental tissues. The oxidative stress was quantified by measuring the extent of lipid peroxidation, the concentration of 8-isoprostane, and catalase activities in tissue samples (Fig. 4g–i). ELISA-based quantification was carried out for Thiobarbituric acid reactive substances (TBARS) and 8-Isoprostane, which are formed as by-products of lipid peroxidation and are quantified instead of reactive oxygen species as they have a very short half-life. TBARS had highest concentration in PE tissues (4.5- 5 nmol/ml, n = 10, p < 0.001) followed by term placental tissues (2.5- 3 nmol/ml, n = 10, p < 0.05) and least in the early villi (1.5–1.8 nmol/ml, n = 10, p < 0.05). The concentration of 8-Isoprostane, a by-product of lipid peroxidation, was also found to be the highest in PE tissues (4.2–4.8 pg/ml, p < 0.001), whereas the term tissues and early villi showed values ranging from 2.5–2.9 pg/ml (p < 0.01) and 1.3–1.7 pg/ml (p < 0.05) respectively. The activity of the antioxidant enzyme catalase (CAT) was also quantified using ELISA, and the highest catalase activity was observed in the healthy term placentas (39–41 nmol/min/ml, p < 0.05) followed by PE tissues (29–32 nmol/min/ml, p < 0.05) and the least activity were found in the early villi tissues^10^ (19–21 nmol/min/ml, p < 0.01).

### H_2_O_2_ induced oxidative stress in trophoblast cells

Based on our observations in placental tissue samples, which showed elevated ROS-related signatures in PE, we performed oxidative stress stimulation on trophoblast cell lines (HTR8/SVneo and BeWo) to obtain the sub-lethal dose of H_2_O_2_ by assessing proliferation and cell viability. Cell lines were treated with different concentrations of H_2_O_2,_ and cell death was monitored using flow cytometry (Suppl Fig. 3 & 4). We performed WST-1 assay and Annexin V-PI staining to quantify the extent of apoptosis. Results of WST-1 assay showed that the sub-lethal dose for BeWo cells was 80 μM H_2_O_2_ (Suppl Fig. 5a.) and for HTR8/SVneo cells was 40 μM H_2_O_2_ (Suppl Fig. 5b). Rescue experiments were done by pretreating the cells 2 h before treatment with H_2_O_2_ using 10 mM *N*-acetyl cysteine (NAC). The experiments were done in four groups, viz—control, H_2_O_2_ only, NAC only, and pretreated group. Annexin-PI staining in HTR8/SVneo cells showed that 78.4 ± 2% of cells were viable in the control group, 62.6 ± 5.2% in the H_2_O_2_ group, 87 ± 2.4% in the NAC group, and 70.4 ± 3.1% in the pretreated group (Supp Fig. 5c). Whereas in BeWo cells 85.9 ± 1.7% of cells were viable in the control group, 75.1 ± 2.2% in the H_2_O_2_ group, 79.5 ± 5% in the NAC group, 78.4 ± 2.1% in the Pretreated group. cells, (Suppl Fig. 5d). Based on the above observation it was found that 80 μM and 40 μM H_2_O_2_ were the chosen sublethal doses for BeWo and HTR8/SVNeo respectively and we measured the H_2_O_2_ mediated oxidative stress.

**Figure 5:**
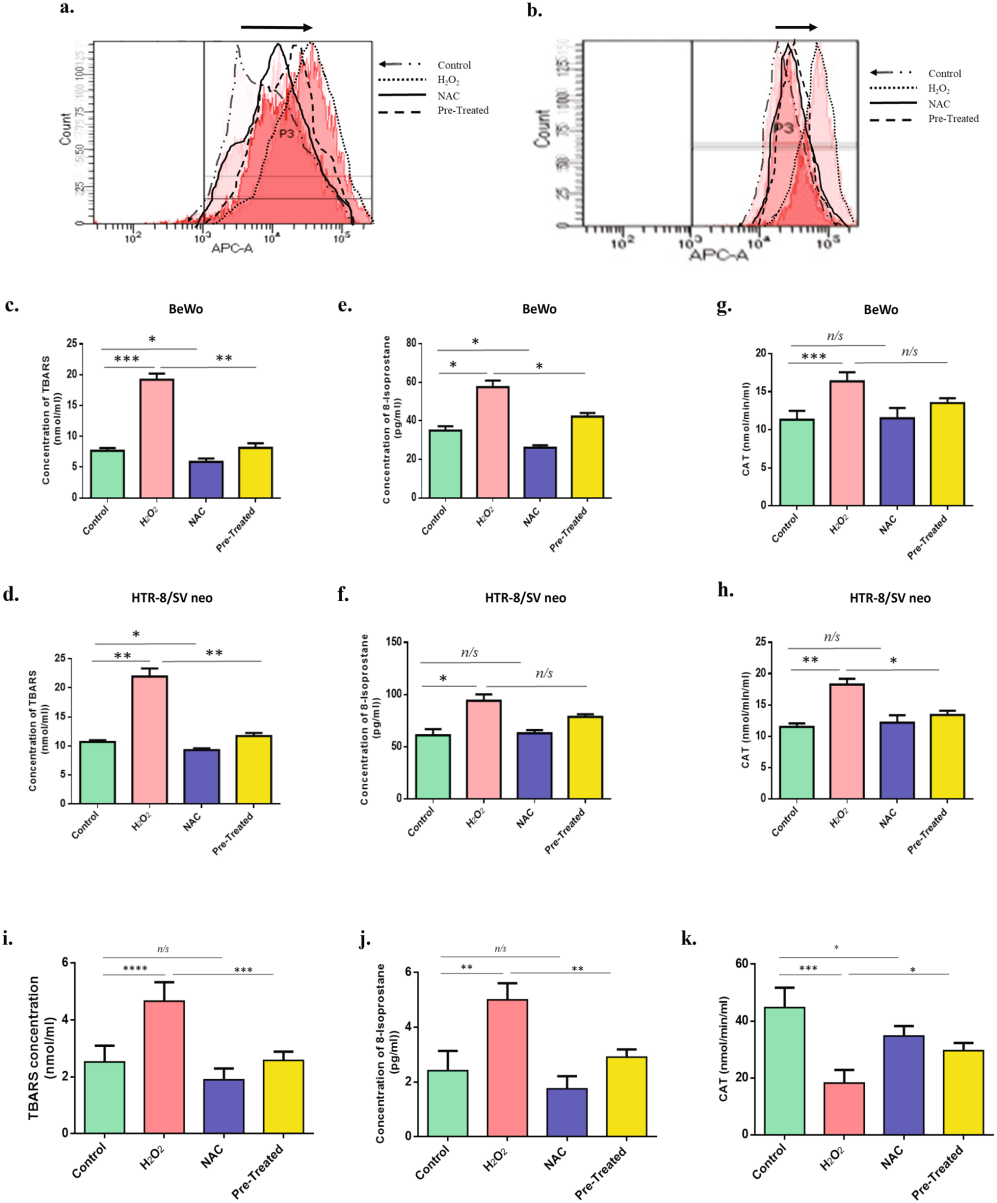
H_2_O_2_ promotes ROS generation in BeWo and HTR8/SVneo cells. ROS production was determined in the untreated, H_2_O_2_, NAC and H_2_O_2_ + NAC (pretreated), stimulated BeWo **(a)** and HTR8/SVneo **(b)** trophoblast cells; n = 3. using Cell ROX Deep Red staining. Data represented as shift in peak with increased ROS production in BeWo and HTR8/SVneo cells; n = 3. False-color was used to outline the peaks of different stimulation groups. ROS levels were also quantifed using oxidative stress markers. Lipid peroxidation product TBARS **(c,d)**, 8-Isoprostane **(e,f)** and antioxidant enzyme Catalase activity **(g,h)** were quantified in untreated, H_2_O_2_, NAC, and H_2_O_2_ + NAC treated BeWo and HTR8/SVneo cells using ELISA, and data was plotted graphically. **(i–k)** TBARS, 8-Isoprostane and Catalase activity were determined in the untreated, H_2_O_2_, NAC and H_2_O_2_ + NAC (pretreated) early placental explants. All data are shown as mean ± standard deviation. Results are representative of at least three independent experiments. *p < 0.05; **p < 0.01; ***p < 0.001; ****p < 0.0001.

Flow-cytometry data of trophoblastic BeWo and HTR-8/SV cells stained with CellRox showed a high level of ROS in the H_2_O_2_-treated group. This can also be seen as a shift in the peak of the treated group compared to the control group (n = 3, p < 0.05). Cells pre-stimulated with H_2_O_2_ and NAC showed no change in peak shift compared to the H_2_O_2_ group, thereby implying a ROS-mediated specific event that was rescued by the antioxidant NAC. (Fig. 5a,b). To further validate our results, we estimated the levels of three oxidative stress markers, TBARS, 8-Isoprostane, and Catalase enzyme activity. Detailed data are shown in Table 1. The concentration of all three markers was significantly higher in H_2_O_2_ treated BeWo and HTR8/SVneo cells compared to the Control. Levels of all stress markers were significantly reduced when cells were pretreated with NAC and followed by H_2_O_2_ treatment (Fig. 5c–h).

**Table 1:** Levels of Oxidative stress markers in BeWo and HTR8/SVneo experimental groups.

Sample	OS markers (HTR8/SVneo)			OS markers (BeWo)			Sample size (n)
	TBARS (nmol/ml) ± SEM	8-Isoprostane (pg/ml) ± SEM	Catalase activity (nmol/mil/ml) ± SEM	TBARS (nmol/ml) ± SEM	8-Isoprostane (pg/ml) ± SEM	Catalase activity (nmol/mil/ml) ± SEM	
Control	10.68 ± 0.17	60.9 ± 3.4	11.51 ± 0.32	7.6 ± 0.25	34.95 ± 1.25	11.33 ± 0.66	3
H_2_O_2_	21.95 ± 0.79 ***	94.06 ± 3.55 **	18.27 ± 0.52 ***	19.2 ± 0.58 ****	57.52 ± 1.96 ***	16.37 ± 0.68 **	3
NAC	9.28 ± 0.17 **	62.99 ± 1.72	12.17 ± 0.69	5.86 ± 0.31 *	26 ± 0.76 **	11.51 ± 0.77	3
Pre-treated	11.7 ± 0.29 ***	78.58 ± 1.48 *	13.4 ± 0.4 **	8.13 ± 0.43 ***	42.14 ± 1.08 **	13.5 ± 0.37 *	3

*p < 0.05; **p < 0.01; ***p < 0.001.

OS's effect on placental explants was also examined by performing similar assays after subjecting the early placental explants to H_2_O_2_ . It was observed that there was an increase in the OS biomarkers in explants except for the CAT activity, which was found to be reduced in the stimulated group (Fig. 5i–k) and comparable to those seen in PE tissues. Based on the OS estimation data obtained from PE tissues, H_2_O_2_ stimulated cell lines, and explants, we could conclude that there is an induction of OS in PE patients' placental cells, which could potentially affect the cellular and other processes and thereby cause adverse effects observed in PE.

### Oxidative stress modulated the expression of differentiation markers and invasion markers in BeWo and HTR-8/SVneo cells

**Figure 6:**
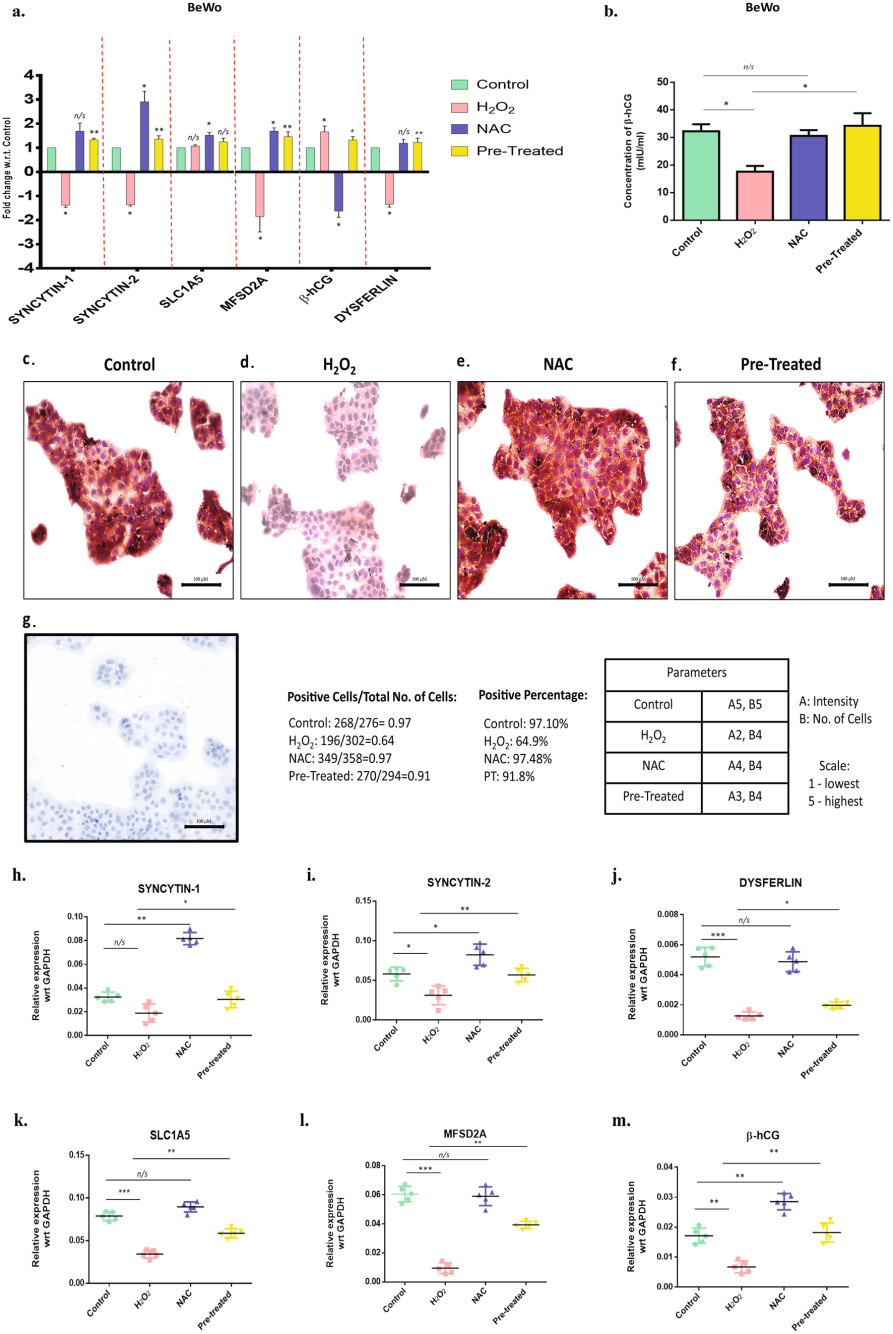
OS mediated decreased differentiation of BeWo cells. **(a)** qPCR was performed to quantify the changes in mRNA expression of the differentiation markers Syncytin-1, Syncytin-2, SLC1A5, MFSD2A, β-hCG and Dysferlin, in the untreated, H_2_O_2_, NAC and H_2_O_2_ + NAC (pretreated), treated BeWo cells; n = 3. Statistical analysis was done by comparing the fold change in H_2_O_2_ & NAC only group with respect to the Control, and the fold change in pretreated cells was compared to the H_2_O_2_. The results were analyzed by the 2^−△△CT^ method (△△CT = △CT value of Treatment -△CT value of another treatment. **(b)** Protein expression of β-hCG was measured in the treatment groups using ELISA. The data was plotted graphically. **(c–g)** Immunocytochemistry was performed in the BeWo cells. Images show the expression of Syncytin-1 after stimulation of oxidative stress. IgG control were shown to validate the Syncytin-1 expression. **(h–m)** mRNA expression of Syncytin-1, Syncytin-2, DYS, SLC1A5, MFSD2A and β-hCG expression were measured in the untreated, H_2_O_2_, NAC and H_2_O_2_ + NAC (pretreated) early placental explant. All data are shown as mean ± standard deviation. Results are representative of at least ten independent experiments. *p < 0.05; **p < 0.01; ***p < 0.001.

qPCR-based quantification showed significant downregulation of the mRNA expression of Syncytin-1, Syncytin-2, SLC1A5, MFSD2A, and Dysferlin (n = 3, p < 0.05) in the H_2_O_2_ stimulated group. Cells that were co-treated with NAC and H_2_O_2_ had no significant changes in mRNA expression of the differentiation markers when compared to control (Fig. 6a). ELISA based quantification showed β-hCG concentration was significantly decreased in the conditioned media of the BeWo cells stimulated with H_2_O_2_ (18–20 mIU/ml, n = 3, p < 0.01) compared to the Control (32–34 mIU/ml, n = 3, p < 0.05) (Fig. 6b). Immunocytochemistry of BeWo cells revealed less expression of Syncytin-1 in the H_2_O_2_ group when compared to control (Fig. 6c–g).

**Figure 7:**
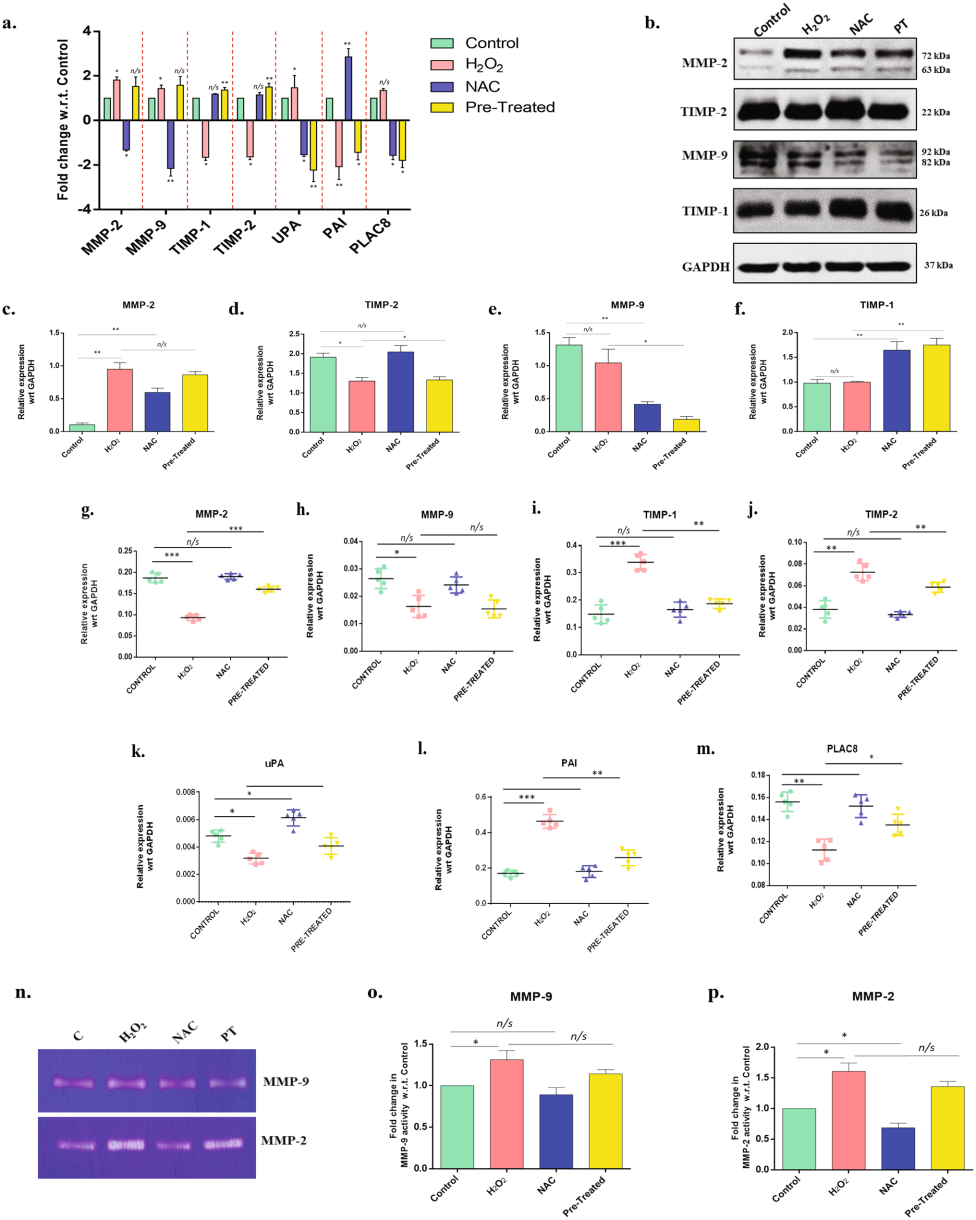
OS alters the level of proteases proteins and their inhibitors **(a)** qPCR was performed to quantify the change in mRNA expression MMP-2, MMP-9, TIMP-1, TIMP-2, UPA, PAI, and PLAC8, in the untreated, H_2_O_2_, NAC, and H_2_O_2_ + NAC (pretreated), treated HTR-8/SVneo trophoblast cells; n = 3. Statistical analysis was done by comparing the fold change in H_2_O_2_ & NAC only group with respect to the Control, and the fold change in Pretreated cells was compared to the H_2_O_2_. The results were analyzed by the 2^−△△CT^ method (△△CT = △CT value of Treatment -△CT value of another treatment. **(b)** Western blotting showing the level of proteases' protein profile and protease inhibitors. MMP-2, MMP-9, TIMP-1, and TIMP-2 antibodies. GAPDH served as the loading control. **(c–f)** Band intensities were quantified and normalized over the GAPDH values. Band intensities were quantified using ImageJ and plotted graphically. **(g–m)** mRNA expression MMP-2, MMP-9, TIMP-1, TIMP-2, UPA, PAI, and PLAC8, in the untreated, H_2_O_2_, NAC, and H_2_O_2_ + NAC (pretreated) early placental explants. **(n–p)** Gelatin Zymography (n = 3) was performed with the cell supernatant from stimulated cells and Control, to observe the activity of MMP-2 and MMP-9. Band intensities were quantified using ImageJ and plotted graphically. Results are representative of at least three independent experiments. *p < 0.05; **p < 0.01; ***p < 0.001.

To confirm that the above observations are not just artifacts from cell line-based experiments, placental explants were also used to study the effect of H_2_O_2_ on trophoblast differentiation. We observed a similar trend in the expression of differentiation markers Syncytin-1 and Syncytin-2, respectively, therefore, validating our previous results. Further, the hCG expression was also observed to increase in the explants after stimulation with H_2_O_2_ in accordance with those seen in the trophoblast cells. (Fig. 6h–m). mRNA expression of invasive markers MMP-2, MMP-9, and uPA was significantly upregulated (n = 3, p < 0.05) in H_2_O_2_ stimulated HTR8/SVneo cells compared to control. Also, TIMP-1, TIMP-2, and PAI expression were significantly downregulated in the H_2_O_2_ stimulated group (n = 3, p < 0.05). Pre-treatment of cells with NAC and H_2_O_2_ showed significant upregulation of MMP-2 and MMP-9 with no change in the expression of TIMP-1 and TIMP-2 (Fig. 7a). Protein levels were also examined for MMP-2, MMP-9, TIMP-1, and TIMP-2. Western blotting data showed significant upregulation of MMP-2 and downregulation of MMP-9 after H_2_O_2_ (n = 3, p = 0.01) stimulation, and the levels were reduced significantly when cells were pre-stimulated with H_2_O_2_ and NAC (Fig. 7b–f). These findings suggest that OS indeed is involved in altering the invasive and differentiation ability of the trophoblast cells.

While H_2_O_2_ stimulation in placental explants resulted in a significant reduction of MMP-2 and MMP-9 along with an upregulation in TIMP-1 and TIMP-2 expression (Fig. 7g–m), similar to what we observed in PE tissues, it disagrees with our results in the trophoblast (HTR8/SVNeo) cells. Although further investigation is required to understand the ambiguity, this disparity is possibly due to the heterogeneity and complexity of placental explants. Pre-treatment with NAC treatment consistently altered the invasion markers in H_2_O_2_ stimulated explants with upregulation of MMP-2 expression and downregulation of TIMP-1 and TIMP-2 expression, suggesting OS mediated modulation of trophoblasts.

### Oxidative stress altered the invasion and migration properties in the HTR-8/SV neo cells

**Figure 8:**
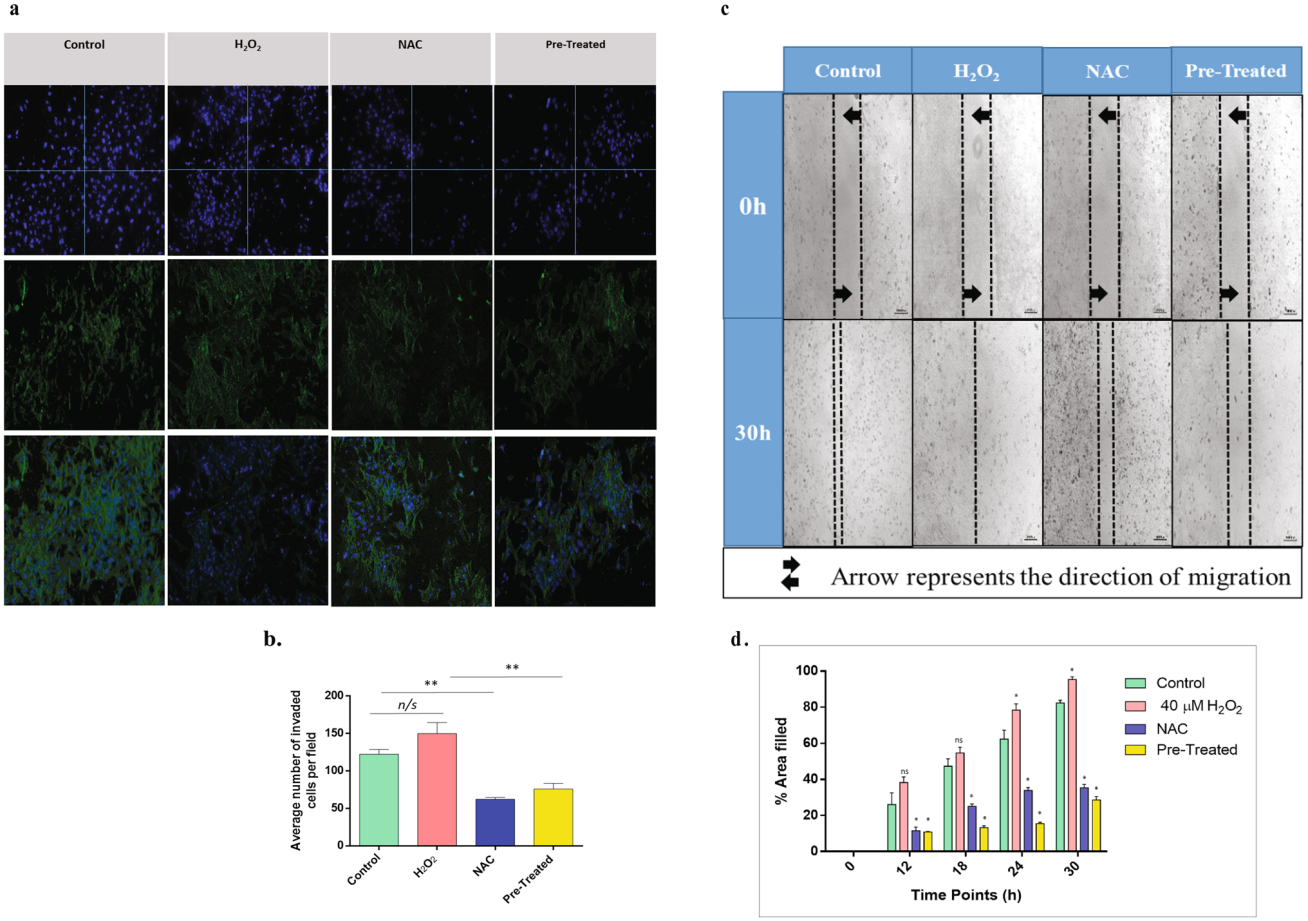
OS mediated decreased invasion and migration of HTR8/SVneo cells. **(a,b)** Matrigel invasion assay was performed to assess the effect of oxidative stress on the invasion property of untreated, H_2_O_2_, NAC, and H_2_O_2_ + NAC (pretreated), treated HTR-8/SVneo trophoblast cells (n = 3). Fluorescence images of invaded cells were taken by staining with DAPI and Phalloidin. The result was quantified by counting the average number of invaded cells in four random fields using ImageJ, and data were plotted graphically. **(c,d)** Wound healing assay was performed (n = 3) to assess the effect of oxidative stress on the migratory behavior of the cells. Even scratches were made, and the healing of the wound was observed for 30 h. The wound area was calculated using ImageJ for every designated time interval. Fold difference in the extent of the area filled was calculated and plotted graphically for each time interval. All results are represented as mean ± standard deviation. Results are representative of at least three independent experiments. *p < 0.05; **p < 0.01; ***p < 0.001.

To further validate the effect of H_2_O_2_ and OS on the invasive and migratory behavior of trophoblast cells by modulating the matrix-degrading enzymes, functional assays like gelatin zymography, Boyden chamber assay, and the Scratch assay were performed. Zymography showed that MMP-2 and MMP-9 significantly upregulated in cells stimulated with H_2_O_2_ (n = 3, p < 0.05). Cells pre-stimulated with H_2_O_2_ and NAC did not affect the secretion of MMP-2 and MMP-9, with levels similar to those of control (Fig. 7n–p). Results of the Matrigel invasion assay showed that the invasion index (fraction of cells migrated as compared to total cells) of the H_2_O_2-_treated cells was 1.2-fold higher compared to control, although no significant difference was observed (Fig. 8a,b). Wound healing/scratch assay performed with HTR-8/SV neo cells showed faster migration of H_2_O_2_-treated cells compared to control (Fig. 8c,d), indicating the oxidative stress-mediated invasion and migration of trophoblast cells.

### Oxidative stress triggers UPR pathway in trophoblast cells: the IREα -XBP1s axis

**Figure 9:**
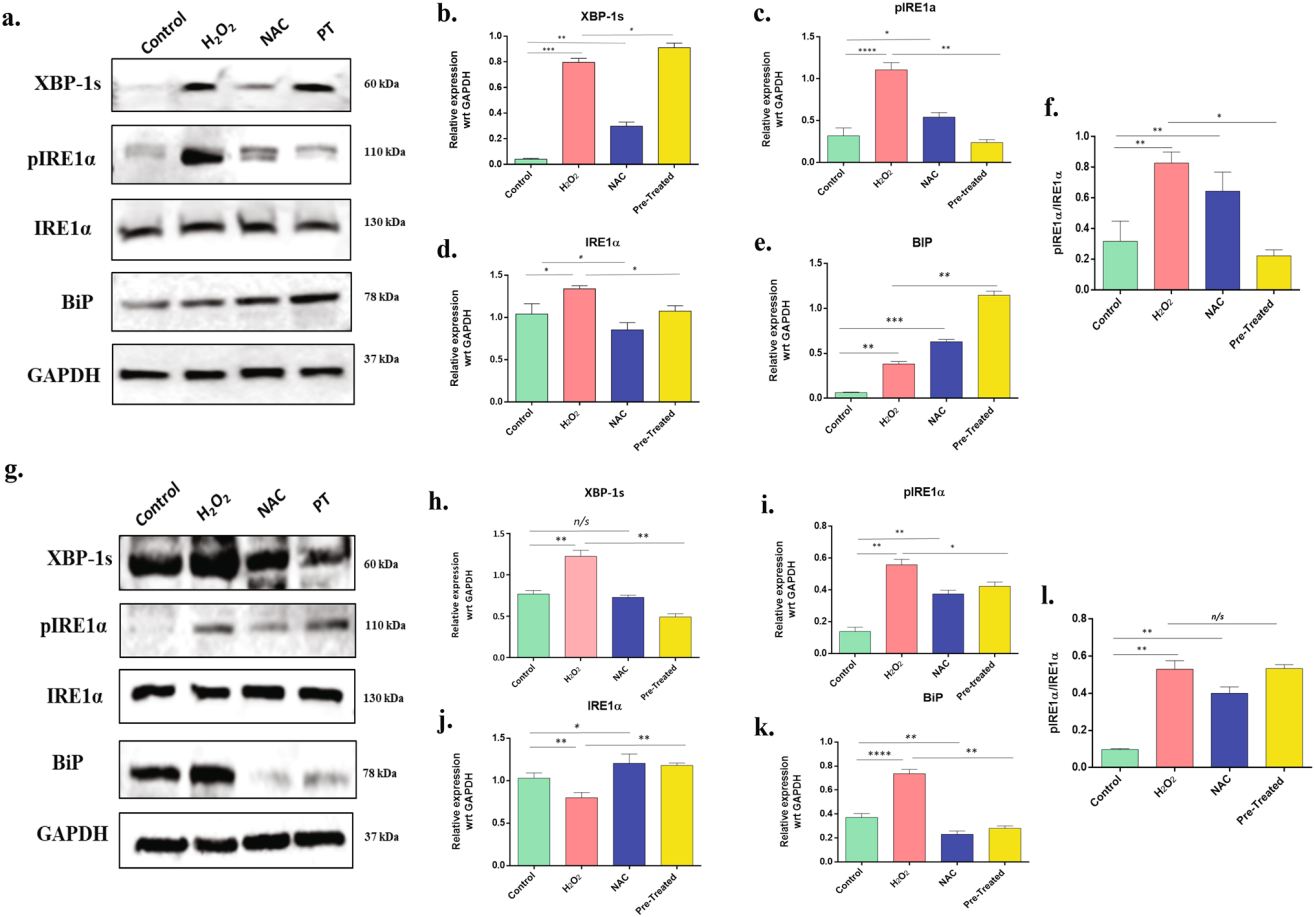
Oxidative stress-induced ER stress through UPR pathway in BeWo and HTR8/SVneo cells. **(a)** Western blotting was performed (n = 3) to assess and quantify the expression proteins involved in UPR pathway. Lysates isolated and prepared from the untreated, H_2_O_2_, NAC, and H_2_O_2_ + NAC (pretreated), treated BeWo cells were immunoblotted for BiP, pIRE1α, IRE1α, XBP-1s. GAPDH served as the loading control. Band intensities were quantified and normalized over the GAPDH values. **(b–e)** Band intensities were quantified using ImageJ and plotted graphically. **(f)** Ratio of pIRE1α**/**IRE1α was calculated in the different treatment groups in BeWo cells **(g)** Lysates isolated and prepared from mentioned four groups of HTR8/SVneo cells (n = 3) were immunoblotted for BiP, IRE1α, XBP-1s. GAPDH served as the loading control. Band intensities were quantified and normalized over the GAPDH values. **(h–k)** Band intensities were quantified using ImageJ and plotted graphically. **(l)** Ratio of pIRE1α**/**IRE1α was calculated in the different treatment groups in HTR8/SVneo cells. All data are shown as mean ± standard deviation. Results are representative of at least three independent experiments. *p < 0.05; **p < 0.01; ***p < 0.001; ****p < 0.0001.

In many pathophysiological conditions, OS and ER stress response are integrated and exist with a mutual cross talk between them^32^. Some studies postulated that OS can be a consequence of ER stress^33^, but few studies have credited the involvement of OS in inducing ER stress and UPR pathway^34^. UPR can profoundly affect various cellular signalings, such as redox homeostasis, inflammation, proliferation, and apoptosis. Under homeostatic conditions, UPR is inactive and is subject to regulation by glucose-regulated protein 78 (GRP78: also known as immunoglobulin heavy chain-binding protein (BiP), which keeps UPR-related proteins from ensuing a UPR^13,16–18,20^. One of the proteins regulated by GRP78 is the inositol-requiring enzyme 1α (IRE1α). IRE1 signaling is the most conserved ER stress sensor-activated early during ER stress and rapidly attenuated. IRE1α autophosphorylation enhanced further oligomerization of the protein to stimulate RNase activity^35^. After activation, pIRE1 induces the cleavage of an mRNA that translates to X-box-binding protein 1 (XBP1). XBP1s being an important transcriptional activator, mediated the expression of many UPR genes^14,16,17^. So, to identify the induction of UPR expression of these three proteins (BiP, pIRE1α IRE1α, and XBP-1s) were measured. Western blotting for these proteins was performed from the lysates of HTR8/SVneo cells and BeWo cells after subjecting them to required experimental conditions. Results of the immunoblot experiments showed that H_2_O_2_-stimulated BeWo cells had significantly upregulated levels of BiP, pIRE1α IRE1α, and XBP-1s with respect to control (Fig. 9a–e), while in HTR-8/SV neo cells, significant upregulation was found in BiP, pIRE1α and XBP-1s (n = 3, p < 0.05–0.0001) (Fig. 9g–k). These results showed that OS induced ER stress in both trophoblast cells. Further, we plotted the ratio of pIRE1α and IRE1α in both the cell lines (Fig. 9f,l) and results showed that OS was significantly increased the expression of the phosphorylated form of IRE1α further confirming the activation of UPR pathway due to induction of oxidative stress in the cells.

## Discussion

Preeclampsia is a disorder of pregnancy often characterized by maternal hypertension and proteinuria developing after 20 weeks of gestation. Affecting 8–10% of all pregnancies worldwide, PE is a multisystem disease with far-reaching consequences^36^. Trophoblasts exposed to a premature hyperbaric maternal oxygen tension often leads to serious complications^37,38^ and unexpected outcome ^2,5^. The oxygen tension in blood bathing the placental floating villi is < 20 mm Hg until 10–12 'weeks' gestation, following which it elevates to 40–80 mmHg throughout the second and third trimesters^38^. The low oxygen tension during the early gestation week is critical for the initiation of placental angiogenesis^39^. However, the physiological set point of oxygen tension and its dynamic oscillations and adjustments per feto-maternal need is compromised in PE. Studies show that oxidative stress produced during placentation might contribute to the pathogenesis of PE^15^. However, additional factors like iron status, vascular function, and inflammation could also influence this process^40^.

**Figure 10:**
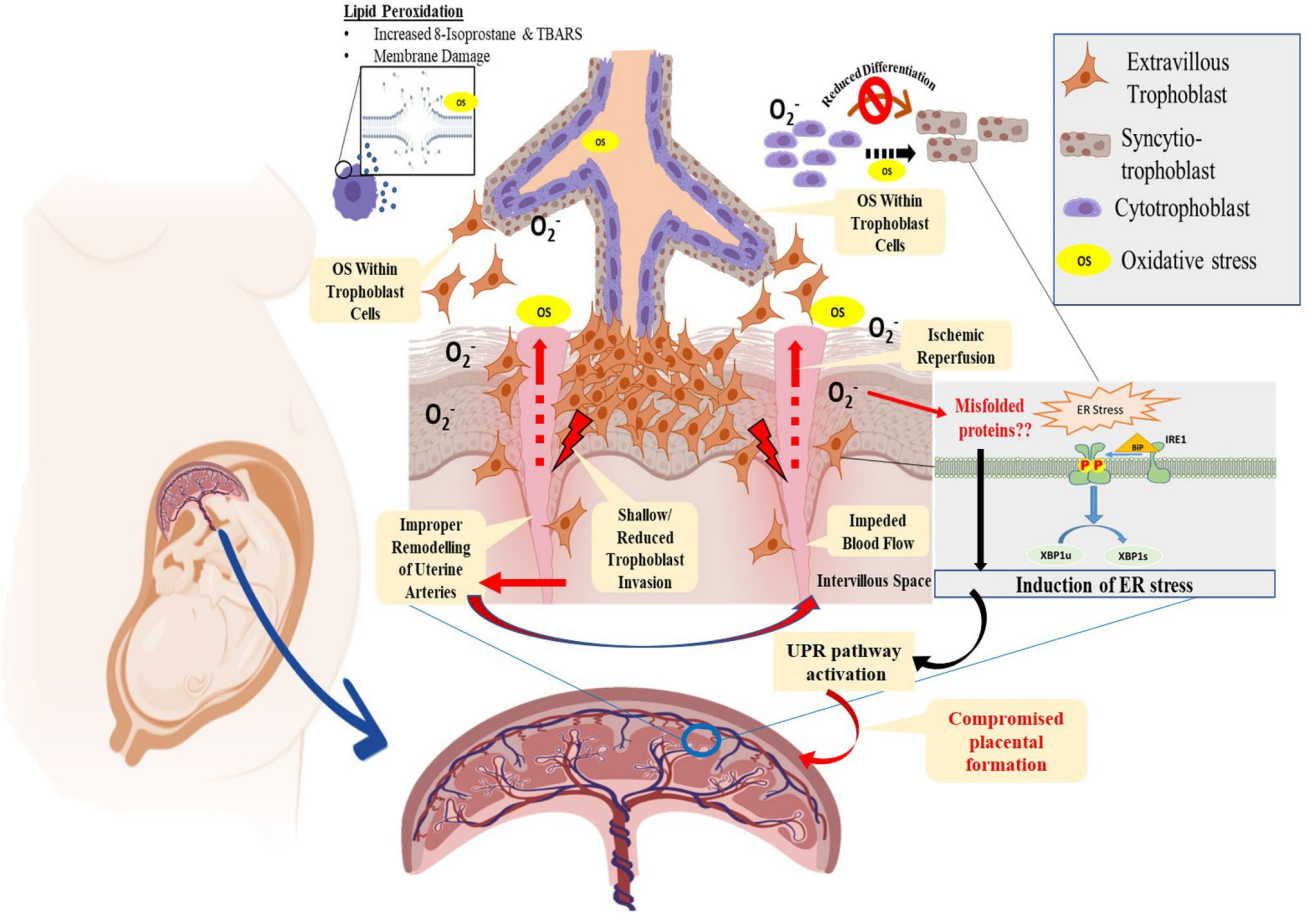
Graphical representation showing OS mediated molecular pathogenesis in preeclampsia where reactive oxygen species (ROS) generated as an outcome of ischemia–reperfusion injury at the feto-maternal compartment originating due to shallow invasion of trophoblast cells. This leads to an inadequate remodeling of uterine spatial arteries, hypoxia as well as an abnormal trophoblast invasion-differentiation program. ROS generation is found to induce oxidative stress leading to an endoplasmic reticulum (ER) injury and protein misfolding by engagement of unfolded protein response (UPR) pathway. Image was drawn by IM and SB.

In this present study, we conclusively identified signatures of oxidative stress in preeclampsia placenta, which prompted us to undertake a detailed methodology-based approach to identify the molecular circuitry driving this process (Fig. 10). Here, we have investigated the role of ROS in the pathogenesis of PE, addressing its effect on cellular and ultrastructural details. The study objectives were accomplished using placental tissues from normal, uncomplicated pregnancies and those of early PE. We have also used immortalized human first-trimester extra-villous trophoblast cells (HTR8/SV neo) and the fusogenic cells (BeWo) in our study to address the effect of external manipulation of conditions. Our analyses identified ROS-mediated trophoblast injury and dysfunction, as confirmed by the increased levels of OS biomarkers such as TBARS, 8-Isoprostane, and catalase activity. Pre-treatment of cells with antioxidant, *N*-acetylcysteine successfully rescued the injury, thereby confirming that the trophoblasts' effects were primarily due to oxidative stress. Mononuclear cytotrophoblasts (CTB) attached to the basement membrane form a syncytial layer by differentiating and fusing to form syncytiotrophoblasts (STB). This cell layer covers the placenta and mediates the exchange of wastes, gases, and nutrients between maternal and fetal blood^41^. OS stress generated in the placenta has the potential to affect the differentiation of trophoblast cells. Our study revealed that the trophoblast differentiation markers expression was significantly low in PE compared to normal placental tissues. The enhanced production of β-hCG also accompanies differentiation of CTBs to STBs^16,18,42,43^ which is also the critical hormone for maintaining pregnancy. This is often called syncytialization, and it involves trophoblast sheet formation that serves as an exchange support system of gas and nutrients between the developing conceptus and the mother^21^. Interestingly, we observed that PE patients had high levels of β-hCG in their serum. Our findings are consistent with some other studies too, indicating that an elevated serum hCG amongst PE subjects who were matched for the same gestational age in normal pregnancy. Though the exact reason is not known, this observation was indeed reported by many researchers^44^. To understand whether there is an involvement of ROS in reduced syncytialization and turn in the formation of STB, we studied the role of the trophoblast fusogenic protein Syncytin, which was previously reported to be associated with trophoblast fusion ^45,46^. Fusogenic program of trophoblast cells to form multinucleated syncytiotrophoblast in the placenta-bearing organisms require the involvement of human endogenous retroviral (hERV) *env* genes that were captured by placenta bearing organisms 12–80 MYA back^47^. Within the human placenta, HERV-W and HERVFRD families encoding syncytin-1 and syncytin-2, respectively, seem to be the most critical active elements^48^. Studies showed that SLC1A5 and MFSD2A are the cognate receptors of Syncytin-1 and Syncytin-2, respectively. These retroviral products are known for their involvement in trophoblast fusion, and altered expression of these genes has been associated with PE ^28,47,49,50^. Our study elegantly elucidated oxidative stress and ROS's effect on the mRNA expression of all the well-established trophoblast differentiation markers and their receptors. Syncytin-1& 2 were downregulated in PE as well as in OS-induced cells. Syncytin-1 is associated with STB formation by CTB fusion, whereas Syncytin-2 is related to the fusion of STB with the STB^50^. Along with Syncytin, we also assessed the levels of β-hCG and Dysferlin in the cells after induction of oxidative stress. Dysferlin is a protein of the ferlin family that contributes to the repair of the damaged syncytiotrophoblast plasma membrane^51^. Thus, increased levels of β-hCG and decreased Dysferlin expression observed in PE patients can be attributed to the generation of OS. Further, it is reported that Syncytin-2 is also associated with the immunosuppressive function^52^, a feature crucial for the survival of a semi-allogenic fetus in the maternal endometrial environment and to avoid maternal immune surveillance. Based on the above report, reduced expression of Syncytin2 in ROS-treated trophoblasts and in the placental explants in our study implies that ROS produced in the placental bed could perturb the immune homeostatic mechanisms. This observation is supported by numerous reports showing a hyperactive immune system^48,53^ driving PE pathogenesis through innate and adaptive immune activation^54,55^.

Trophoblast invasion into the stroma of the endometrium and up to one-third of the myometrium is one of the critical processes for a healthy pregnancy in humans^21^. Accumulating information suggests that regulated MMP activity at the feto-maternal interface is essential for successful placentation. Trophoblasts are highly invasive due to their capacity to produce MMPs^56,57^. Regulated trophoblast invasion and proper vascularization are mainly attained due to the synergy between the proteases and their inhibitors^56^. Our results show that the PE tissues have low expression of proteases like MMP-2, MMP-9 & uPA, and high expression of protease inhibitors such as TIMP-1, TIMP-2, and PAI^37^. PLAC8 is a newly identified gene expressed in the interstitial extravillous trophoblasts (iEVT) that promotes invasion and migration. We also assessed the expression of PLAC8, in the placental tissues, and results showed significant downregulation of PLAC8 in PE tissues. Similar results were obtained when HTR8/SVneo and BeWo cells stimulated with H_2_O_2_, resulting in a reduced differentiation and invasion. These results may explain the reasons behind the shallow invasion observed in PE. Further, we propose that EVT populations that invade the endometrium express Syncytin2, enabling them to avoid from an immune-mediated killing ^58^. ROS-mediated reduction in Syncytin2 could potentially kill these cells by maternal immune surveillance, thereby preventing them from vascular remodeling, as observed in PE. We further speculated that an altered redox state in the cells could epigenetically silence the expression of trophoblast differentiation inducers^59^.

Transmission electron microscopy revealed considerable ultrastructural abberations in PE subjects, which might partially be attributed to an increased ROS production. The decrease in microvillar density in the PE placenta may indicate hampered trophoblast maturation that leads to a decreased metabolic exchange^60–62^, which was further supported by the presence of the larger amount of glycogen in PE tissues. Mitochondrial disintegration may be initiated by ROS-induced peroxidation of its membrane lipids, resulting in a change in mitochondrial membrane potential. This may result in the opening of mitochondrial permeability transition pores, which could lead to release of proapoptotic proteins^58,63^. The presence of disintegrated mitochondria in the PE placenta may indicate ROS-generated OS and cellular assault thereby disintegrating trophoblast’s intracellular components. OS mediated mitophagy also regulate mitochondrial dysfunction^64^, but recent finding showed a decreased mitophagy in the placentas of PE mice^65^. However, though the causes of mitochondrial dysfunction and mitophagy in PE are still unknown but these chain of events could lead to ER stress, a feat that is well documented in PE ^66–68^. ER stress triggers several protective response mechanisms. However, the prolonged activity of these pathways may lead to apoptosis ^15^. The presence of swollen endoplasmic reticulum in PE placenta might be due to OS insult of placental cells. This may further explain the reason behind the trophoblastic dysfunction during PE.

Therefore, we propose that oxidative stress, which is related to the pathophysiology of PE and induced cell death in trophoblasts. However, our results show that, it also enhanced the invasion and migration of trophoblasts. These apparently contradicting observation is due to the multiple roles of ROS in cellular physiology. ROS can promote cell invasion, cell cycle progression as well as cell migration due to the engagement of several transcription factors (NF-κB, AP-1, p53, HIF-1α, PPAR-γ, β-catenin/Wnt, and Nrf2) as well as promoting inflammation which are known to induce cellular invasion. An excess of ROS or misbalance between antioxidants and ROS may lead to cell death, apoptosis or necrosis. An excess of trophoblast apoptosis may lead to PE^69^. Also the release of placental apoptotic fragments into the maternal circulation promotes endothelial dysfunction and hypertension as reported in PE^70^.

During stressful conditions, luminal domains of the cell's proximal sensors are occupied by BiP/GRP78, an ER HSP70 family protein, which represses UPR pathways ^16,17^. Once ER stress sets in the cells, there is an activation of these sensors by one of the unfolded proteins IRE1α, one of the proximal sensors^13^. IRE1α activation with phosphrylation (pRE1α) results in splicing of Xbp1 mRNA to Xbp1s. This Xbp1s further activates the transcription of the genes involved in ER-associated degradation^13^. In our present study, the activation of the UPR pathway in OS-insulted trophoblasts and PE tissues via the IRE1α -Xbp1s axis may indicate OS-induced ER stress in trophoblasts. This underpins the altered architecture of PE placenta as a result of apoptotic death of trophoblast cells. Whether ER stress directly affects trophoblastic differentiation and invasion properties may further be explored in a detailed mechanistic approach.

Therefore to summarise, our finding suggests that ROS generated within the trophoblast cells leads to an initiation of a chain of events resulting in altered trophoblast function (invasion and differentiation). This also results in UPR leading to cellular damage and ultrastructural changes of intracellular components that further lead to the loss of trophoblast function. ROS-mediated loss of Syncytin1 and 2 may also reduce trophoblast cell fusion and maternal immune evasion. PE placenta shows the signature of ROS-mediated cellular damage and features consistent with oxidative stress insult. Thus, our study provides a framework of mechanistic details that could help us to understand PE pathogenesis in light of these new findings. The information obtained from our study may also be exploited in preventive measures and clinical management of PE.

## Methods

### Patient samples

A total of 30 placental samples of different gestational ages were obtained from patients with PE (n = 10), medically terminated first trimester pregnancies (n = 10), and normotensive pregnancies (n = 10) after delivery from Department of Obstetrics & Gynaecology, All India Institute of Medical Sciences (AIIMS), New Delhi. All procedures were carried out as per approval and guidelines (IEC-225/05.04.2019) by Institute Ethics Committee, AIIMS. New Delhi. Informed written consent was obtained from all patients enrolled in this study. The blood samples of the patients were collected two days before delivery. The serum soluble fms-like tyrosine kinase-1 (sFlt-1 or sVEGFR) levels in the pregnant women with PE and the healthy normotensive women were measured using sFlt-1 ELISA kit (Cayman Chemicals, CA, USA). Clinically diagnosed PE patients with elevated serum sFLT-1 levels were considered for placenta collection. Sample validation data have been given in Suppl. Figure 6a. PE was characterized with patient blood pressure higher than 140/90 mmHg and elevated proteinuria (≥ 300 mg/24 h urine collection), serum creatinine levels greater than 1.1 mg/dL, and platelets < 100,000 µl (in the absence of proteinuria). The diagnosis of PE was based on strict criteria from the American College of Obstetricians and Gynecologists (ACOG 2013). All of the patients whose sFLt-1 levels were higher than the threshold levels of the control were ruled out for any pre-existing underlined hypertensive disorders. Patient characteristics are shown in Table 2. All of the placental tissue samples in the study were obtained immediately after delivery and processed fresh. Sections of placental tissue (1 × 1 × 1 cm) were taken from the four different central quadrants on the maternal face of the placenta. After washing with sterile 1×  phosphate-buffered saline (PBS), tissues were minced and immediately kept in TRIzol and RIPA buffer separately until used for RNA and protein extraction, respectively, and stored at −80 °C. Some sections of the tissues were also held in RIPA buffer for Thiobarbituric Acid Reaction Substances (TBARS) and 8-Isoprostane assay and in phosphate buffer for catalase assay. Sections of the villi (1 × 1 × 1 mm) were cut and fixed in Karnovsky's fixative for Electron Microscopy studies and stored at 4 °C.

**Table 2:** Patient characteristics. Details of the patients from whom blood, urine and placenta samples were collected.

Parameters	EP (10)	Control (10)	PE (10)
Maternalage	21 ± 3	30 ± 4	31 ± 2
Gestational age of delivery (wk)	8 ± 2	37 ± 2	35 ± 2
Gestational age at first elevatedBP (wk)	–	–	26 ± 4
Systolic BP (mm Hg)	120 ± 2	124 ± 4	138 ± 2
Diastolic BP (mm Hg)	74 ± 5	72 ± 6	100 ± 8
Proteinuria(mg/dl)	–	–	900 ± 50
Placentalweight (g)	20 ± 5	450 ± 100	350 ± 100
Fetalweight (g)	–	3000 ± 130	2300 ± 160

### Cell culture

BeWo (choriocarcinoma cells) and HTR-8/SV neo cells were obtained from American Type Culture Collection (ATCC, Rockville, MD, USA). Cells from early passages (R = 3) were cultured in RPMI-1640 medium (HyClone) containing 10% FBS (Invitrogen) and 1% penicillin–streptomycin (Invitrogen). The cells were plated on a 10 cm petri dish at a concentration of 1 × 10^6^ cells/petri dish and kept at 37 °C in a humidified chamber with 5% CO. For experiments, cells were divided into four groups—(i) control, (ii) cells treated with H_2_O_2_ for 24 h, (iii) cells treated with N-acetyl cysteine (NAC) for 24 h, and (iv) cells pretreated with NAC for 2 h followed by H_2_O_2_ treatment for another 24 h. At 50–60% confluence, cells were kept in phenol red-free medium containing 5% FBS and treated with NAC and/or the sub-lethal dose of H_2_O_2_. Sample validation with sFLT-1 expresssion have been given in Suppl. Figure 6b, c. Cells were then scraped and processed for RNA and protein extraction.

### Explant culture

Tissue explants from early villi (n = 5) were cultured. Small sections weighing 10 mg were cut and plated in Collagen-I coated single well of a 12 well plate (Corning). The tissue sections were washed thoroughly using 1×  phosphate-buffered saline (PBS) followed by the addition of RPMI-1640 medium (HyClone) containing 10% FBS (Invitrogen) and 1% penicillin–streptomycin (Invitrogen). The well plate was then kept at 37 °C in a humidified chamber with 5% CO_2_. For experiments, sections from each early villi were divided into four groups—(i) control, (ii) sections treated with H_2_O_2_ for 24 h, (iii) sections treated with *N*-acetyl cysteine (NAC) for 24 h, and (iv) sections pre-treated with NAC for 2 h followed by H_2_O_2_ treatment for another 24 h (Suppl. Figure 7).

### RNA isolation and reverse transcription‑quantitative polymerase chain reaction (RT‑qPCR)

Total RNA was extracted from the placental villous tissues, explants, and cells using an RNA Simple Total RNA kit (Promega RNA Tissue/Cell Miniprep System) according to the manufacturer's instructions. RNA was quantified using Nanodrop (Thermo Fisher Scientific, Inc.), and agarose gel electrophoresis was performed to check the integrity of the isolated RNA. The isolated RNA was reverse transcribed into cDNA using the Verso cDNA synthesis kit (AB1453A, Thermo Fisher Scientific, Inc.). RT-qPCR was performed using DyNAmo Flash SYBR Green qPCR kit (F415S, Thermo Fisher Scientific, Inc.). The RT-qPCR reaction cycles were as follows: incubation at 95 °C for 7 min, 40 cycles at 95 °C for 15 s, and 60 °C for 20 s. The PCR products were subjected to a melting curve analysis to confirm the amplification specificity. Levels of mRNA were normalized with respect to GAPDH mRNA levels and evaluated using the 2-ΔΔCq method^10^. PCR primer sequences have been provided in the Suppl. Table 1.

### ROS production

Cells were plated in 12-well plates. After treatment of cells, as mentioned above, 5 μM CellRox (Cellular ROS Assay Kit, ab186029S) was added to each well and incubated at 37 °C for 30 min. The cells were then washed 3 times with 1X PBS at room temperature and analyzed by flow cytometry using BD FACSCanto™.

### Quantification of oxidative stress markers

Thiobarbituric Acid Reaction Species Assay (Cayman Chemicals) was performed to determine Malondialdehyde (MDA) levels, the final product of lipid peroxidation. The concentrations of thiobarbituric acid reactive substances (TBARS) were measured in cells and the collected tissue samples, explants according to the manufacturer's protocol. The concentration of 8-Isoprostane was measured in the cells and tissues using an assay kit (Cayman Chemicals, USA) following the manufacturer's protocol. The activity of Catalase was assayed in cells and tissues using a catalase assay kit according to manufacturer's protocol (Cayman Chemicals, USA).

### Apoptosis assay

Apoptosis assays were performed by flow cytometry using Annexin V-FITC/propidium iodide double-stain assay, according to the manufacturer's protocol (BMS500FI/100CE, Invitrogen). The treated cells (1 × 10^6^) were collected by trypsinization and incubated in 100 μL binding buffer containing 5 μL AnnexinV/FITC and 10 μL 20 μg/mL PI at room temperature in dark condition for 15 min. 10,000 events were acquired, and results were analyzed using FlowJo software (Ashland, OR, USA).

### Western blotting

Placental tissues and cell lysate were prepared using RIPA buffer supplemented with protease and phosphatase inhibitors. 50 µg of lysates were electrophoresed by 10% SDS-PAGE for western blot (WB) analysis and transferred to polyvinylidene difluoride membrane. Following the transfer, the membrane was blocked in 5% non-fat milk for 1 h. Thereafter the membrane was incubated with the mentioned primary antibodies overnight at 4 °C. The membrane was washed thrice in TBST and then incubated with an appropriate HRP-conjugated secondary antibody for 1 h at room temperature. Proteins were detected using the enhanced chemiluminescence (ECL) solution (NCI4106; Thermo Fisher Scientific, Inc.). Rabit polyclonal anti-IRE1α, rabbit polyclonal anti-BiP, rabbit polyclonal anti-XBP-1s, rabbit polyclonal anti-MMP-2, rabbit polyclonal anti-MMP-9, rabbit polyclonal anti-TIMP-1, and rabbit polyclonal anti-TIMP-2, all from Cell Signal Technology, Beverly, MA were used at 1:1000 dilution in 5% BSA solution containing 0.1% TBST and kept at 4 °C overnight. Rabbit polyclonal Anti-pIRE1α was used from Novus Biologicals, Centennial, USA at 1:1000 dilution in 5% BSA solution containing 0.1% TBST. Finally, the immunoreactive signals were quantified using a densitometer. The same membranes were used for re-probing with an antibody against glyceraldehyde-3-phosphate dehydrogenase (GAPDH) (Immunotag) or Tubulin (Santa Cruz Biotechnology, Texas, US) at 1:1500 dilution.

### Transmission electron microscopy

Placental tissues (1 mm^3^/piece) or cell pellets (10^6^) were fixed with Karnovsky's fixative at 4 °C, rinsed with 0.1 M phosphate buffer (pH ~ 7.4) twice, and post-fixed with 1% osmium tetroxide for 1 h. Samples were then dehydrated in acetone, embedded in Araldite CY212, and polymerized. Sections (60–70 nm thick) were cut and stained with uranyl acetate and lead citrate. Sections were examined using a Talos 200S transmission electron microscope (Thermo Scientific, Inc.) at All India Institute of Medical Sciences. To understand the effect of OS on subcellular component, counting of swollen ER and disintegrated mitochondria was performed and plotted using histogram per 100 sq μm of the EP, TP and PE tissues. The counting was performed in every fifth section that was retrieved during ultramicrotomy. Five sections were utilised for the counting of the number of mitochondria and swollen ER in tissue samples (n = 10) in each group. The counting was performed in a fixed area of 100 sq μm (10 μm × 10 μm) on digital images acquired at a fixed magnification, using ImageJ software.

### Gelatin zymography

Matrix metalloproteinase (MMP)-2/9 activities in the conditioned media of HTR-8/SVneo cells was measured using gelatin zymography. Proteins were resolved using 8% polyacrylamide gel containing 0.1% gelatin. The gel was then washed with 2.5% Triton X-100 solution for 1 h to renature the proteins, followed by washing with distilled water and incubation in activation buffer (150 mM NaCl, 10 mM CaCl_2_, 50 mM Tris, and 0.025% sodium azide) with gentle shaking overnight. Gels were stained with 0.1% Coomassie brilliant blue for 30 min followed by de-staining with 10% acetic acid solution to visualize.

### Wound healing assay

Cells were seeded in six-well plates at a density of 2 × 10^5^ cells per well and cultured until they reached 80–90% confluence. A pipette tip made a scratch through the cell monolayers, and the debris was removed with 1× PBS. Cells were treated as mentioned previously. After 24 h of treatment, microscopic images were captured at regular intervals until complete wound healing (filling up of the scratch with cells).

### Matrigel invasion assay

The invasion assay was performed using a 24-well insert system (8 μm pores; Transwell chamber, Millipore). The insert plate surface was coated with 50 μl of diluted matrigel (356234; Becton Dickinson and Company, Franklin Lakes, New Jersey, USA, 1:9 in RPMI-1640). HTR8/SVneo cell suspension (1.0 × 10^5^ cells) of 200 μl was seeded into the insert, and culture medium supplemented with FBS was added to the reservoir well. Cells were treated and cultured for 24 h. After incubation, the trophoblast cells remaining in the upper insert were removed using a swab, and the trophoblast cells infiltrated and reached the other side of the insert were fixed with ice-cold methanol and stained with 4′,6-Diamidine-2′-phenylindole dihydrochloride (D9564, Sigma-Aldrich) for nuclear staining Images were taken using an upright microscope (TI-E, 601869, Nikon Laser Scanning Confocal Microscope) at 200× magnification.

### Immunocytochemistry

BeWo cells were grown on coverslips coated with 2% 3-Aminopropyl triethoxysilane. Cells were cultured on the coverslips and treated with the required stimulant; following treatment; the cells were fixed with 4% paraformaldehyde followed by membrane permeabilization using 0.1% Triton-X 100 in 1× PBS. Cells were washed with PBS and blocked with 5% BSA solution. Cells were then overnight incubated with rabbit polyclonal anti-Syncytin-1 antibody (Immunotag; dilution: 1:1500). Cells were then washed three times with 1× PBS and incubated with HRP-conjugated anti-rabbit secondary antibody (dilution: 1:1000) at room temperature for 2 h. The cells were washed three times with 1× PBS, air-dried, and mounted using DPX. The slides were visualized using an upright Microscope (TI-E, 601869, Nikon Laser Scanning Confocal Microscope).

### Immunohistochemistry

Tissue samples were washed with PBS and fixed overnight in 4% buffered formalin at room temperature. Paraffin embedment was done, and 5 µm thick sections were cut. The sections were deparaffinized, rehydrated, and microwaved for 20 min in Tris–EDTA buffer (pH 9.0) to retrieve antigens and blocked with 3% H_2_O_2_ for 15 min. Non-specific reactivity was blocked using BlockAid™ Blocking Solution (Invitrogen). Sections were incubated with rabbit anti-Syncytin-1 antibody (dilution: 1:1000; ab3484; Immunotag) at 4 °C in a humid chamber for 12–14 h. Sections were then incubated with fluorophore-conjugated anti-rabbit secondary antibody (DAKO, Santa Clara, USA) at room temperature for one hour, washed thrice with 0.1 M TBS, and mounted with DPX mounting medium. The sections were observed under a fluorescence microscope, using appropriate filter sets.

### Statistical analysis

All experiments were repeated at least three times. Data were presented as mean ± SD. The statistical significance of the results was assessed by Student's t-test using the Graphpad Prism software package (version 5.0; La Jolla, California, USA) with a P value less than 0.05 being considered significant.

## Supplementary Information

Supplementary Information.
