# Supplementary material for: Oxidative stress-induced impairment of trophoblast function causes preeclampsia through the unfolded protein response pathway
Source: Sci Rep. 2021 Sep 16;11:18415. doi: 10.1038/s41598-021-97799-y (PMC8446002; doi:10.1038/s41598-021-97799-y)
Supplement: Supplementary file 1 — Supplementary Information. [file 41598_2021_97799_MOESM1_ESM.pdf]

Title: Oxidative stress-induced impairment of trophoblast function causes preeclampsia through the unfolded protein response pathway

Indrani Mukherjee, Ruby Dhar, Sunil Singh, Jay Bhagwan Sharma, Tapas Chandra Nag, Asit Ranjan Mridha, Parul Jaiswal, Subhrajit Biswas*, Subhradip Karmakar*

Contents:

1. **Additional Methods involved in Supplementary Figures**
2. **Figure Legends for Suppl. Fig. Suppl. Fig. 1 to Suppl. 7**
3. **Suppl. Table 1. Details of primers used in this study.**
4. **7 Suppl. Figures (Suppl. Fig.1 to Suppl. Fig. 7)**
5. **Original Western Blots**

SUPPLEMENTARY DETAILS

Methods

Protocol for early villi explant culture: Tissue explants from early villi (n=5) were cultured. Small sections weighing 10 mg was cut and plated in Collagen-I coated single well of a 12 well plate (Corning).The tissue sections were washed thoroughly using 1X phosphate buffered saline (PBS) followed by addition of RPMI-1640 medium (HyClone) containing 10% FBS (Invitrogen) and 1% penicillin-streptomycin (Invitrogen). The well plate was then kept at 37°C in a humidified chamber with 21% O_2 and 5% CO_2.

For experiments, sections from each early villi was divided into four groups- (i) control, (ii) sections treated with H_2O_2 for 24 h, (iii) sections treated with N-acetyl cysteine (NAC) for 24 h and (iv) sections pre-treated with NAC for 2 h followed by H_2O_2 treatment for another 24 h.

Legends:

Fig 1. Early villi (10 weeks), term placenta (38 weeks) and Preeclampsia placenta (36 weeks) were collected from the patients and measured for dimensions. PE placenta (Fig. 1c) was smaller in size compared to term placenta (Fig 1b). Early villi (Fig 1a) tissue was well branched.

Fig 2a. Patient samples were screened by checking the level of the biomarker of Preeclampsia. sFlt-1 concentration was measured in the blood serum of the normal pregnant women (NP) and Preeclamptic women (PE) collected two days prior to delivery. **Fig 2(b, c).** Concentration of sFlt-1 was also measured in the conditioned media of the trophoblast cells (BeWo & HTR8/SVneo) treated with H_2O_2. All data are shown as Mean ± Standard deviation. Results are representative of at least twelve independent experiments. * $p < 0.05$; ** $p < 0.01$; *** $p < 0.001$ (Student's t-test).

Fig 3a-b. Images of trophoblast cells HTR8/SVneo & BeWo respectively were taken after the cells were treated with different dose of H_2O_2 viz. 20 μM, 40 μM, 80 μM, 100 μM, 200 μM. The images were taken art 100X Magnification. Each experiment was repeated three times.

Fig 4. Flow-cytometric analysis was done to determine the sub lethal dose of H_2O_2 in the trophoblast cells. A tabular representation of the data has been given. All data are shown as Mean ± Standard deviation. Results are representative of at least three independent experiments. * $p < 0.05$; ** $p < 0.01$; *** $p < 0.001$ (Student's t-test).

Fig. 5. Effect of H_2O_2 on the viability of BeWo and HTR8/SVneo cells. **(a-b)** WST-1 and Annexin-FITC-PI staining were used to assess and quantify the viability of cells. Cells were treated with different doses of H_2O_2 to induce oxidative stress in the cells. WST-1 assay was performed with BeWo and HTR8/SVneo cells to check the sub-lethal dose. **(c-d)** Cell death was measured by flow cytometry using Annexin V-FITC and PI double staining. BeWo and HTR8/SVneo cells were exposed to the chosen concentrations of H_2O_2. In each density plot quadrant Q1: shows necrotic cells (Annexin− PI+); Q2: late apoptotic cells (Annexin+ PI+); Q3: shows the viable cells (Annexin− PI−) and Q4: early apoptotic cells (Annexin+ PI−) Results are representative of at least three independent experiments..

Fig 6. Protein expression of Caspase 3 and PARP was assessed using western blotting. The data was analysed using ImageJ. All data are shown as Mean ± Standard deviation. Results are representative of at least three independent experiments. * $p < 0.05$; ** $p < 0.01$; *** $p < 0.001$ (Student's t-test).

Fig 7. The early villi explants plated on Collagen-I coated well plate have been shown. Four different wells have been considered four different treatment groups.

Suppl.Table 1. Details of primers used in this study.

S.No	Genes	Forward Primer (5′ – 3′)	Reverse Primer (5′ – 3′)	Amplicon Size (bp)
**1**	SYN-1	CTGTTGGACTTACTTCACCCAAA	GGTACGGAGGGTTTCATGTAGT	169
**2**	SYN-2	AGCCCCTATTTGTGTTATGGC	GGAATTGGTTGTGGGTGTATGT	134
**3**	MFSD2A	CCATTGATGAGGAGAGGCGG	CCTTCTGTGGCCTTCTGCAT	162
**4**	SLC1A5	TCATGTGGTACGCCCCTGT	GCGGGCAAAGAGTAAACCCA	186
**5**	DYSF	AAGAACAGCGTGAACCCTGTA	CCTCTCGGAGTGGGACCTT	157
**6**	β-hCG	CTACTGCCCCACCATGACCC	GCAGAGTGCACATTGACAGC	172
**7**	MMP-2	ATGACAGCTGCACCACTGAG	ATTTGTTGCCCAGGAAAGTG	174
**8**	MMP-9	CTCGAACTTTGACAGCGACA	GCCATTCACGTCGTCCTTAT	187
**9**	TIMP-1	CATTGCTGGAAAACTGCAGGA	GCAGTTTGCAGGGGATGGAT	167
**10**	TIMP-2	GGCTGCGAGTGCAAGATCAC	TCGAGAAACTCCTGCTTGGG	197
**11**	UPA	CCCAGGAAATGGGACAGGG	ACAGTTCGCCTGTTCGTATCT	199
**12**	PAI	CATCCTGGAACTGCCCTACC	AGGGAGAACTTGGGCAGAAC	174
**13**	PLAC8	GGAACAAGCGTCGCAATGAG	AAAGTACGCATGGCTCTCCTT	152
**14**	GAPDH	ACGGATTTGGTCGTATTGGG	CGCTCCTGGAAGATGGTGAT	214

Suppl. Fig. 1

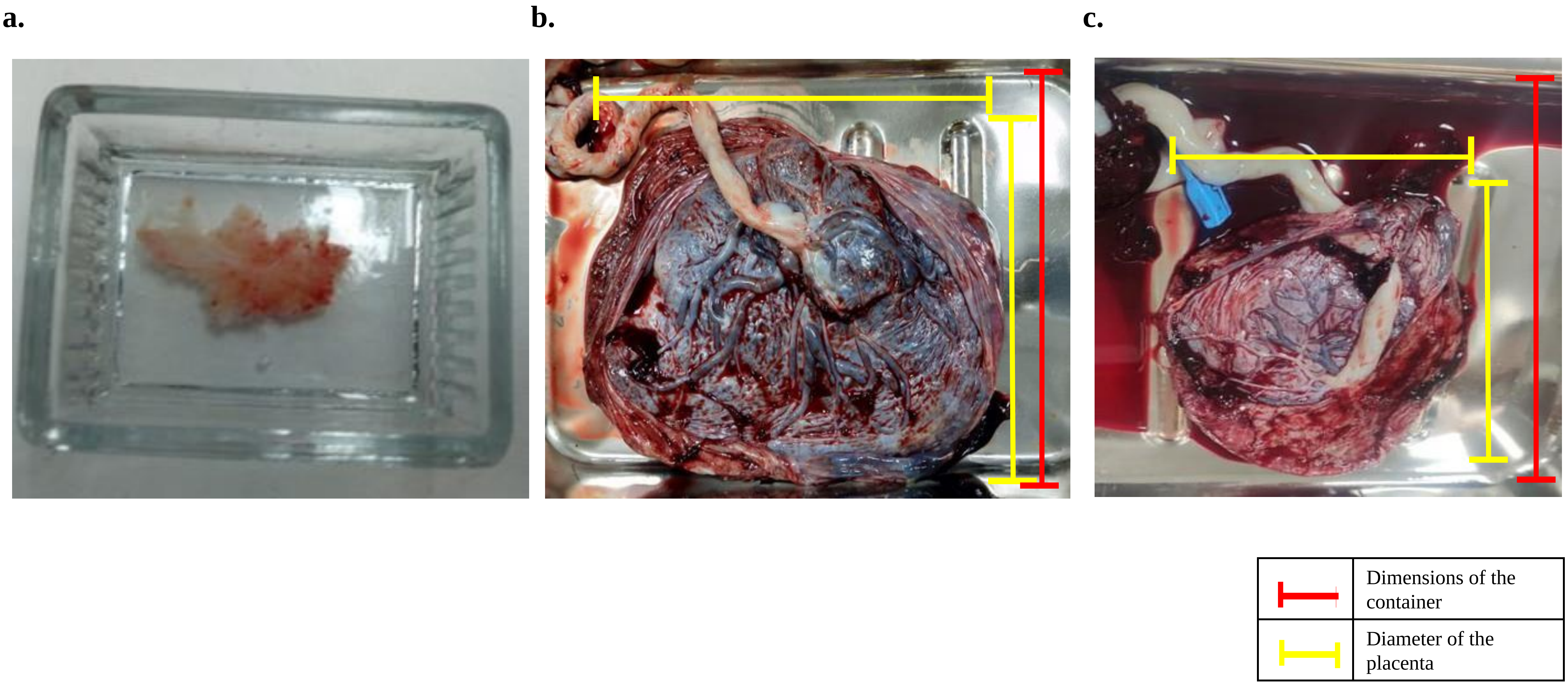

Suppl. Fig. 2

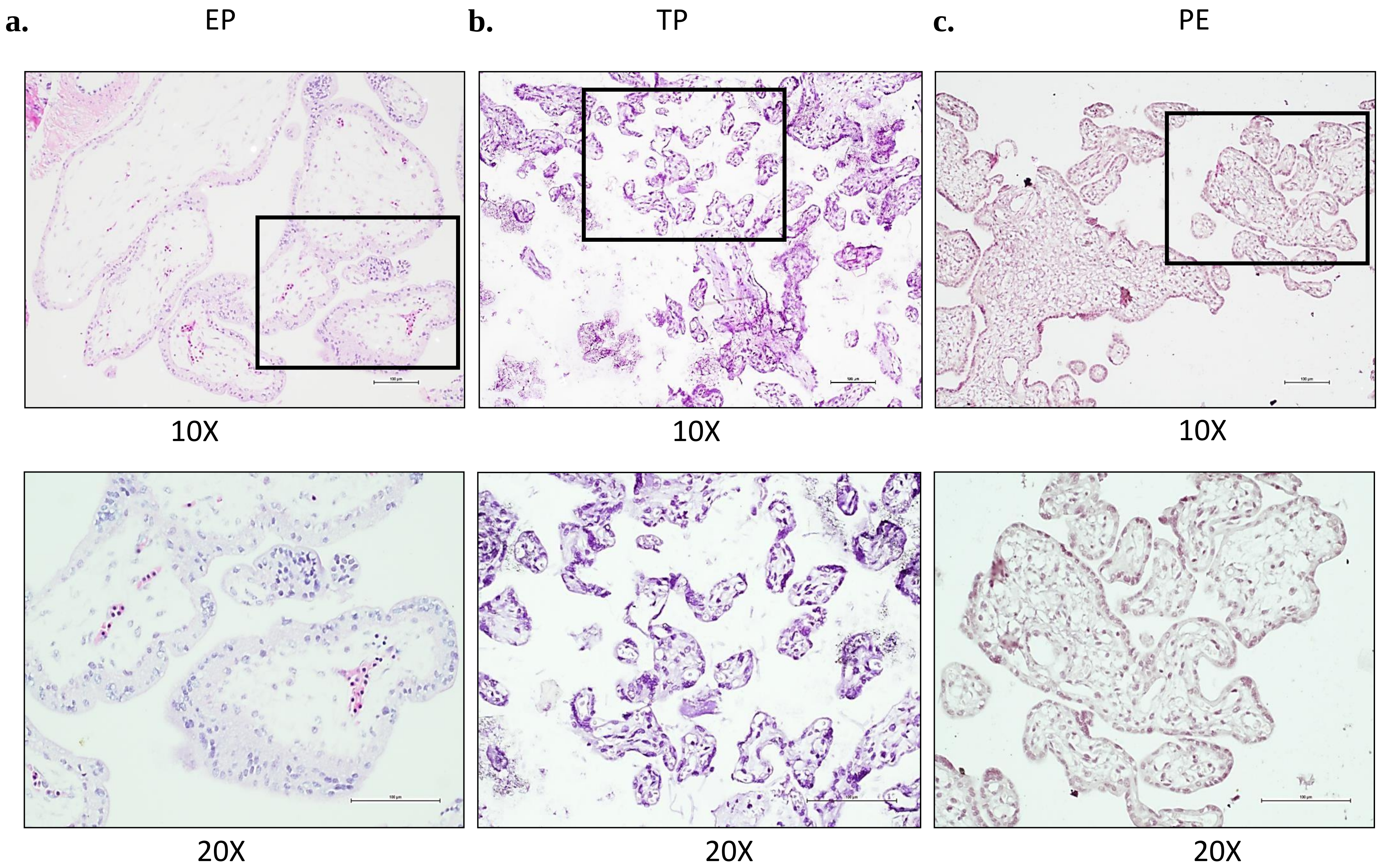

Suppl. Fig. 3

a.

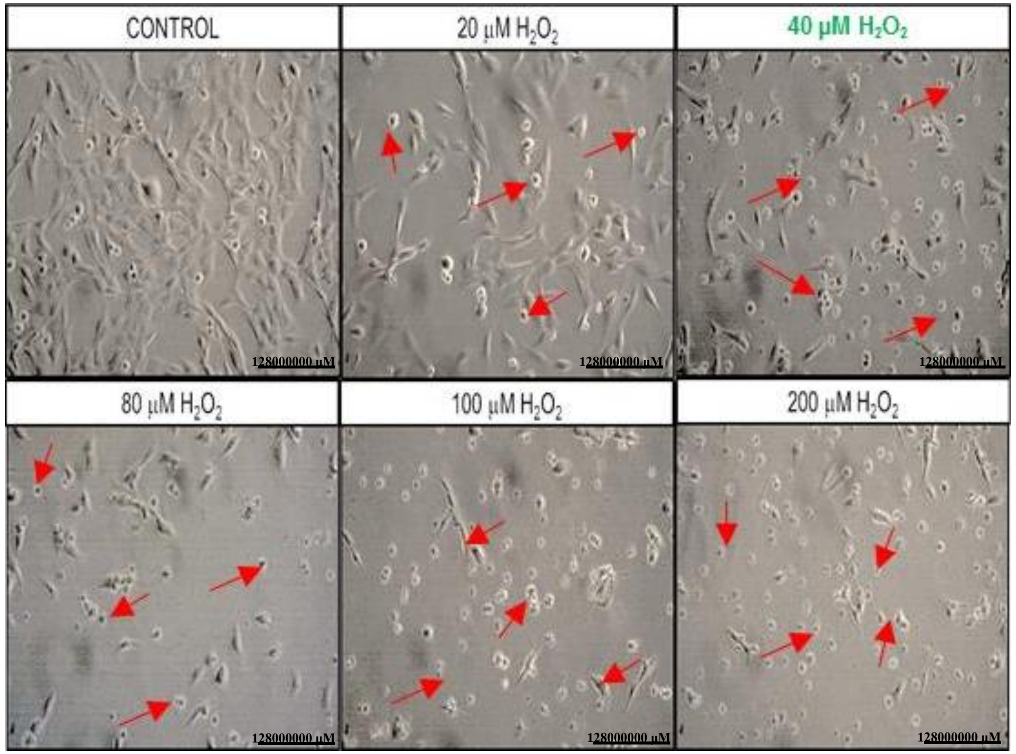

n=3; Magnification: 10X

b.

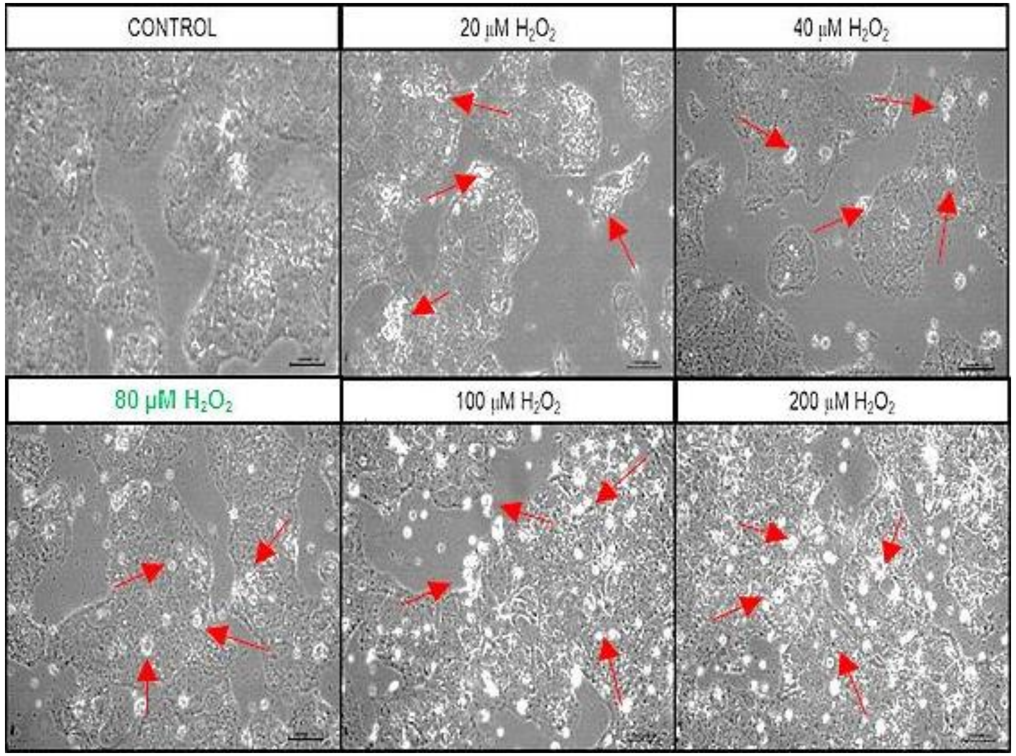

n=3; Magnification: 10X

Suppl. Fig. 4

a.

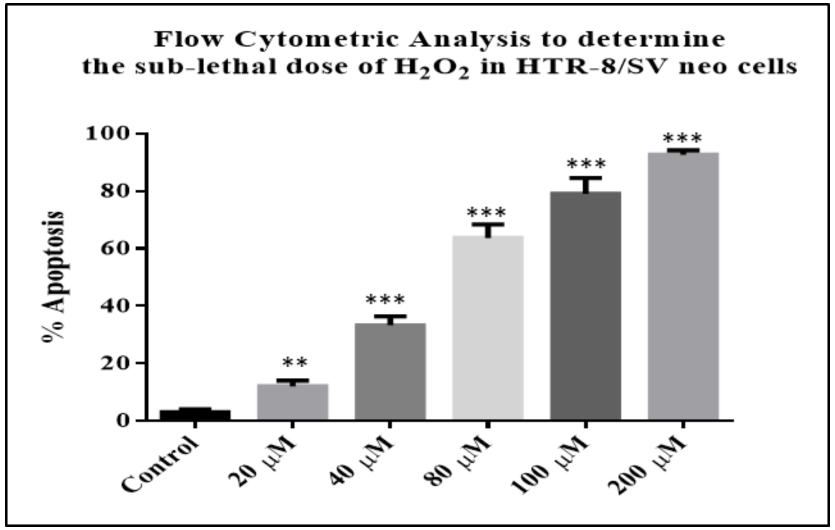

b.

Treatment Groups in HTR-8/ SV neo cells	Percent Apoptosis **
Control	2±1
20 µM H_2O_2	12±2 **
**40 µM H_2O_2**	**34±2** ***
80 µM H_2O_2	62 ± 5 ***
100 µM H_2O_2	87 ±7 ***
200 µM H_2O_2	92 ± 5 ***

c.

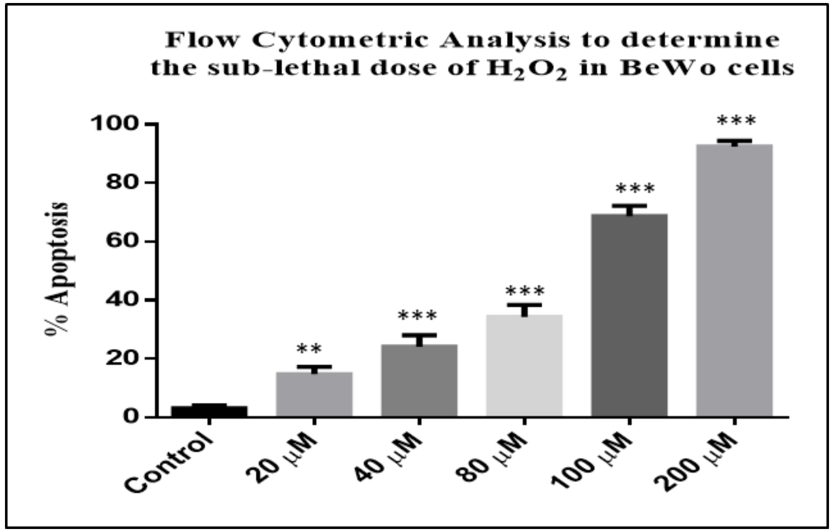

d.

Treatment Groups in BeWo cells	Percent Apoptosis
Control	2±1
20 µM H_2O_2	12±2 **
40 µM H_2O_2	24 ±2 ***
**80 µM H_2O_2**	**38 ± 5***
100 µM H_2O_2	69 ±7 ***
200 µM H_2O_2	90±9 ***

Suppl. Fig. 5

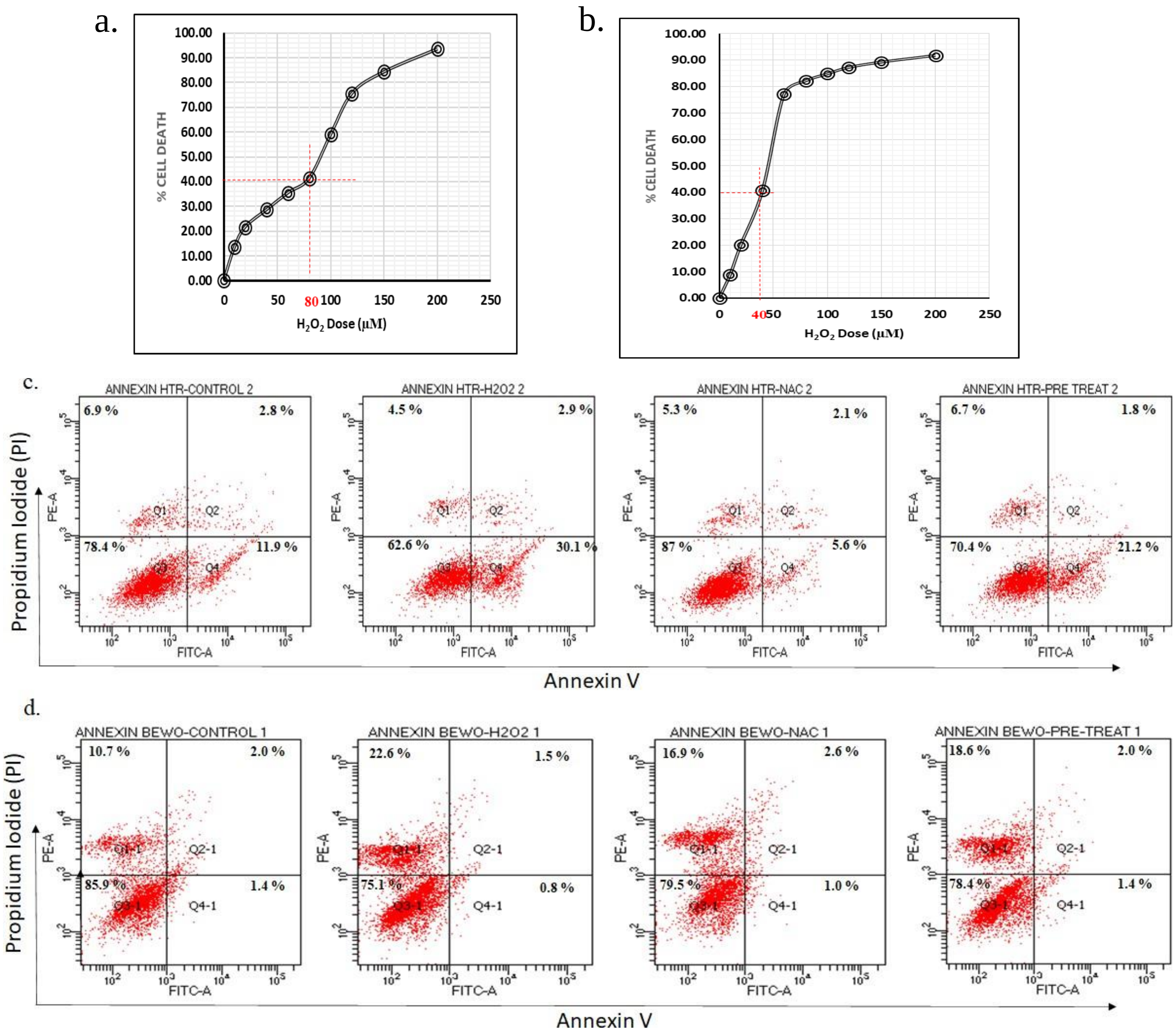

a.
% CELL DEATH
H2O2 Dose (µM)
80
b.
% CELL DEATH
H2O2 Dose (µM)
40
c.
ANNEXIN HTR-CONTROL 2
ANNEXIN HTR-H2O2 2
ANNEXIN HTR-NAC 2
ANNEXIN HTR-PRE TREAT 2
6.9 % 2.8 % 78.4 % 11.9 %
4.5 % 2.9 % 62.6 % 30.1 %
5.3 % 2.1 % 87 % 5.6 %
6.7 % 1.8 % 70.4 % 21.2 %
Propidium Iodide (PI)
PE-A
FITC-A
Annexin V
d.
ANNEXIN BEWO-CONTROL 1
ANNEXIN BEWO-H2O2 1
ANNEXIN BEWO-NAC 1
ANNEXIN BEWO-PRE-TREAT 1
10.7 % 2.0 % 85.9 % 1.4 %
22.6 % 1.5 % 75.1 % 0.8 %
16.9 % 2.6 % 79.5 % 1.0 %
18.6 % 2.0 % 78.4 % 1.4 %
Propidium Iodide (PI)
PE-A
FITC-A
Annexin V

Suppl. Fig. 6

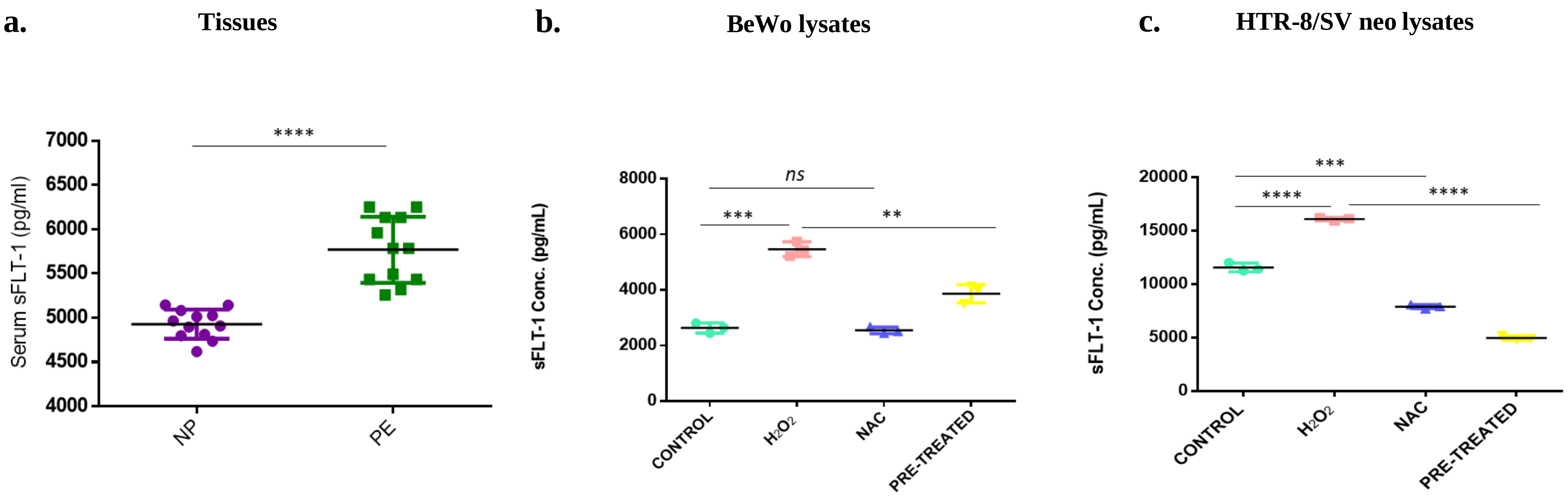

a.
Tissues
****
7000
6500
6000
5500
5000
4500
4000
Serum sFLT-1 (pg/ml)
NP
PE
b.
BeWo lysates
ns
***
**
8000
6000
4000
2000
0
sFLT-1 Conc. (pg/mL)
CONTROL
H2O2
NAC
PRE-TREATED
c.
HTR-8/SV neo lysates
***
****
****
20000
15000
10000
5000
0
sFLT-1 Conc. (pg/mL)
CONTROL
H2O2
NAC
PRE-TREATED

Suppl. Fig. 7

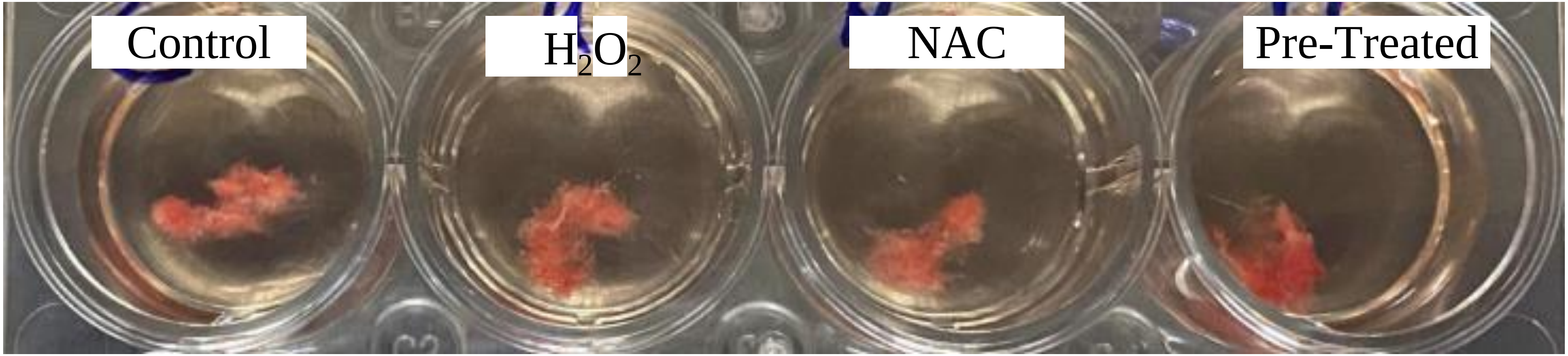

Original Blots

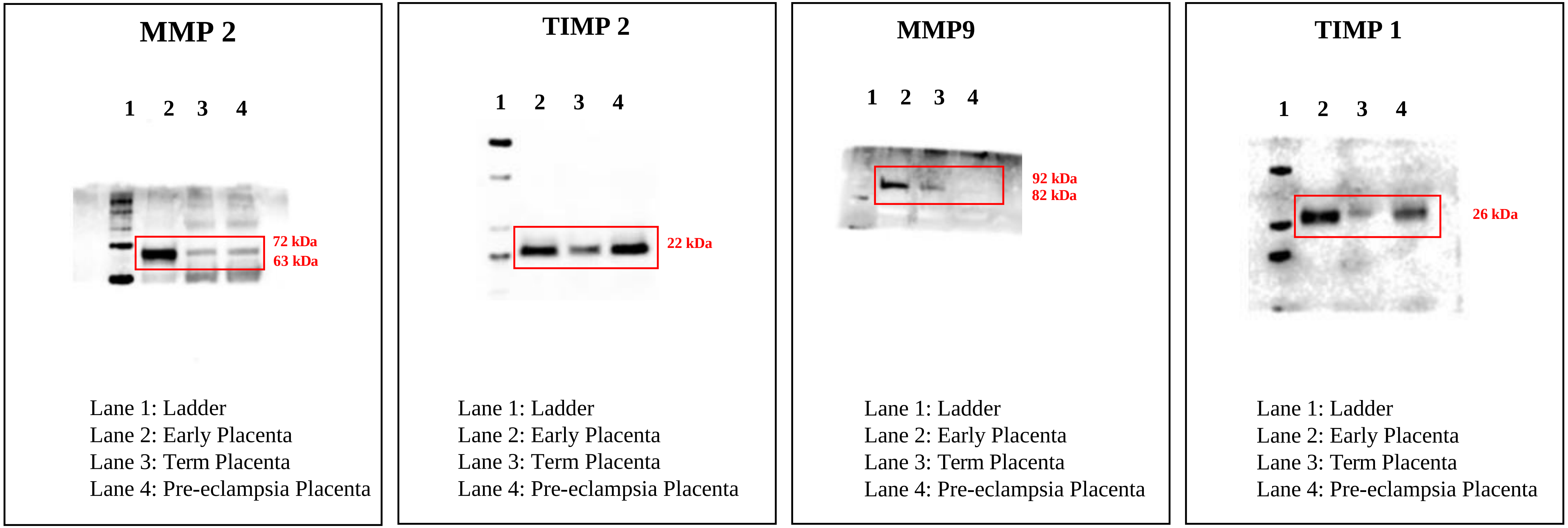
MMP 2
1 2 3 4
72 kDa
63 kDa
Lane 1: Ladder
Lane 2: Early Placenta
Lane 3: Term Placenta
Lane 4: Pre-eclampsia Placenta
TIMP 2
1 2 3 4
22 kDa
Lane 1: Ladder
Lane 2: Early Placenta
Lane 3: Term Placenta
Lane 4: Pre-eclampsia Placenta
MMP9
1 2 3 4
92 kDa
82 kDa
Lane 1: Ladder
Lane 2: Early Placenta
Lane 3: Term Placenta
Lane 4: Pre-eclampsia Placenta
TIMP 1
1 2 3 4
26 kDa
Lane 1: Ladder
Lane 2: Early Placenta
Lane 3: Term Placenta
Lane 4: Pre-eclampsia Placenta

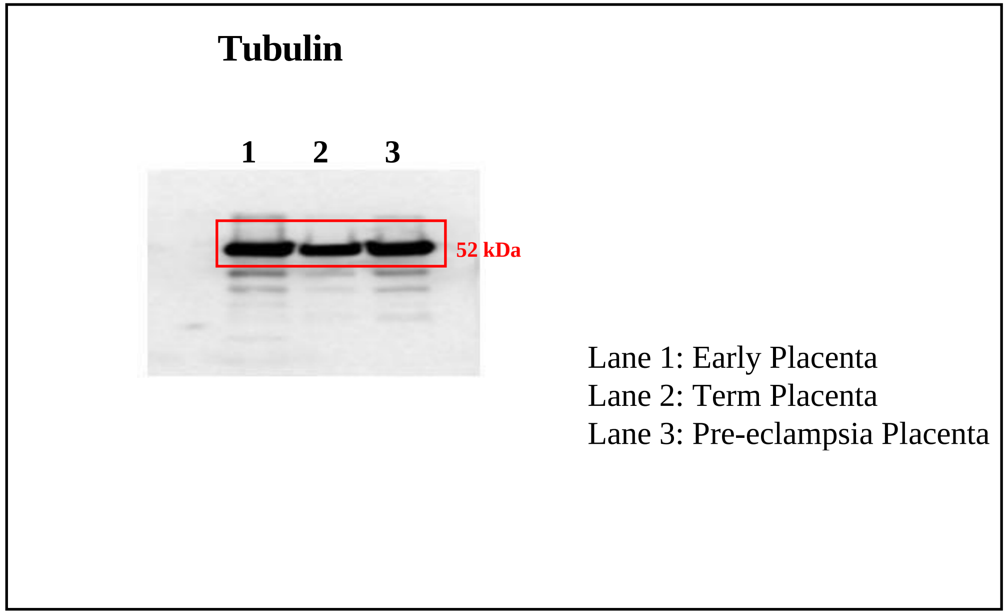
Tubulin
1 2 3
52 kDa
Lane 1: Early Placenta
Lane 2: Term Placenta
Lane 3: Pre-eclampsia Placenta

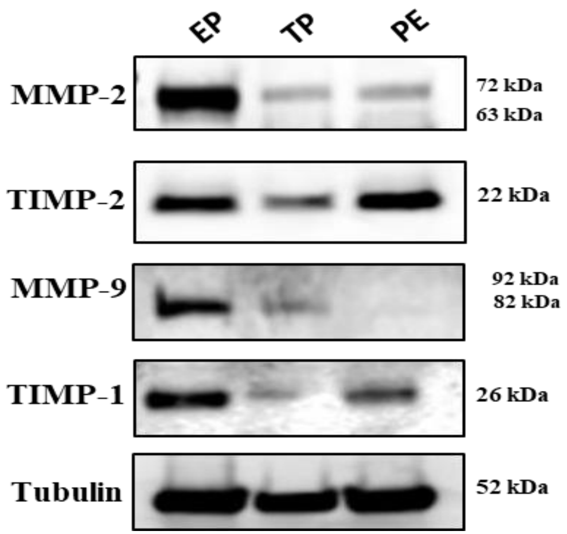
EP
TP
PE
MMP-2
72 kDa
63 kDa
TIMP-2
22 kDa
MMP-9
92 kDa
82 kDa
TIMP-1
26 kDa
Tubulin
52 kDa

Figure 2h.

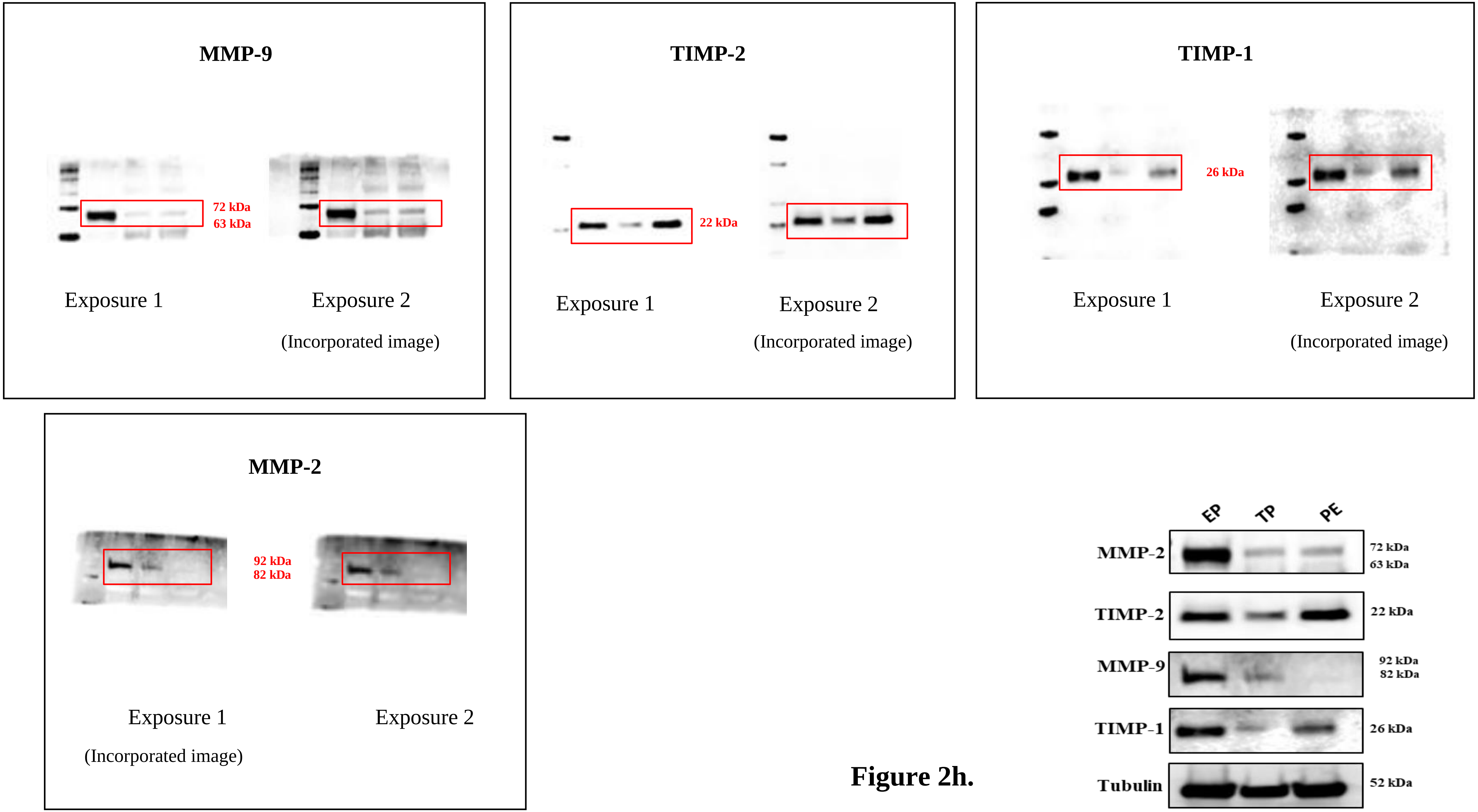

MMP-9
72 kDa
63 kDa
Exposure 1
Exposure 2
(Incorporated image)
TIMP-2
22 kDa
Exposure 1
Exposure 2
(Incorporated image)
TIMP-1
26 kDa
Exposure 1
Exposure 2
(Incorporated image)
MMP-2
92 kDa
82 kDa
Exposure 1
(Incorporated image)
Exposure 2
EP
TP
PE
MMP-2
72 kDa
63 kDa
TIMP-2
22 kDa
MMP-9
92 kDa
82 kDa
TIMP-1
26 kDa
Tubulin
52 kDa

Figure 2h.

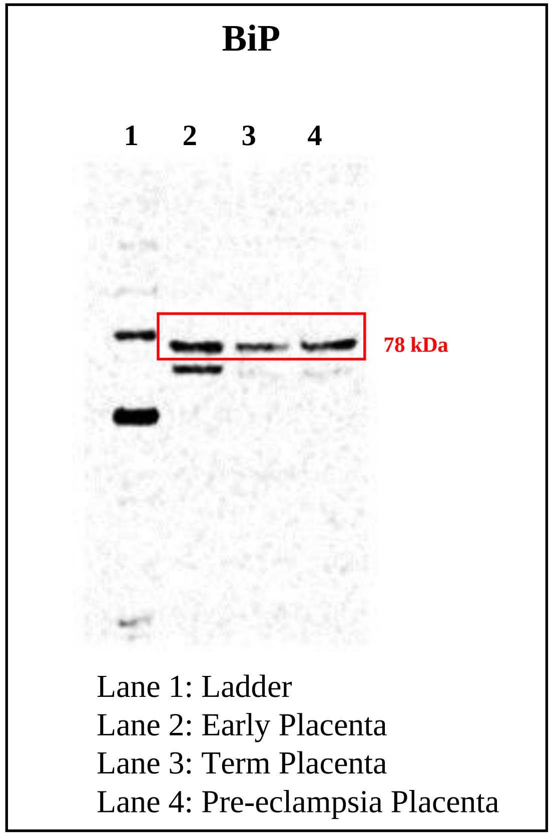
BiP
1 2 3 4
78 kDa
Lane 1: Ladder
Lane 2: Early Placenta
Lane 3: Term Placenta
Lane 4: Pre-eclampsia Placenta

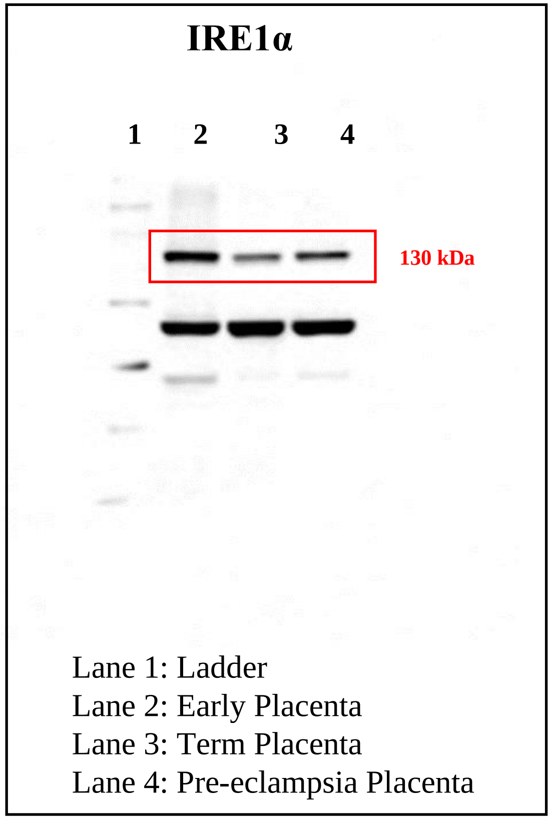
IRE1α
1 2 3 4
130 kDa
Lane 1: Ladder
Lane 2: Early Placenta
Lane 3: Term Placenta
Lane 4: Pre-eclampsia Placenta

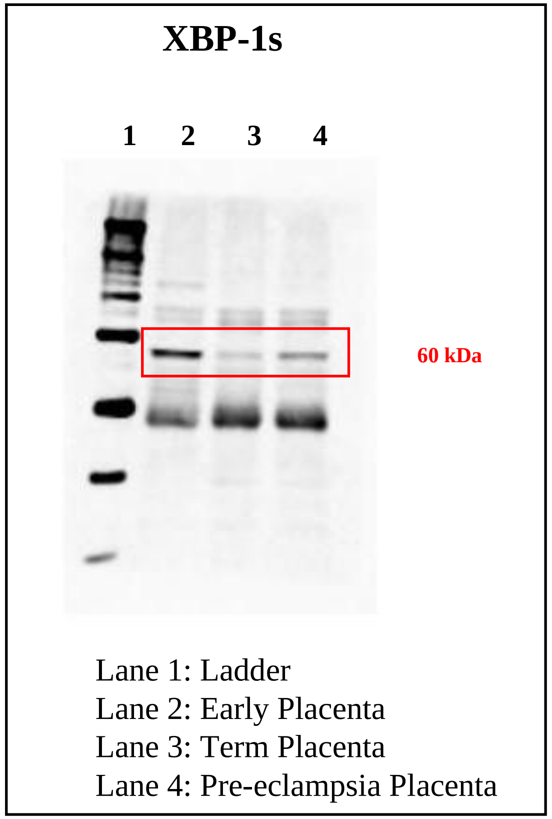
XBP-1s
1 2 3 4
60 kDa
Lane 1: Ladder
Lane 2: Early Placenta
Lane 3: Term Placenta
Lane 4: Pre-eclampsia Placenta

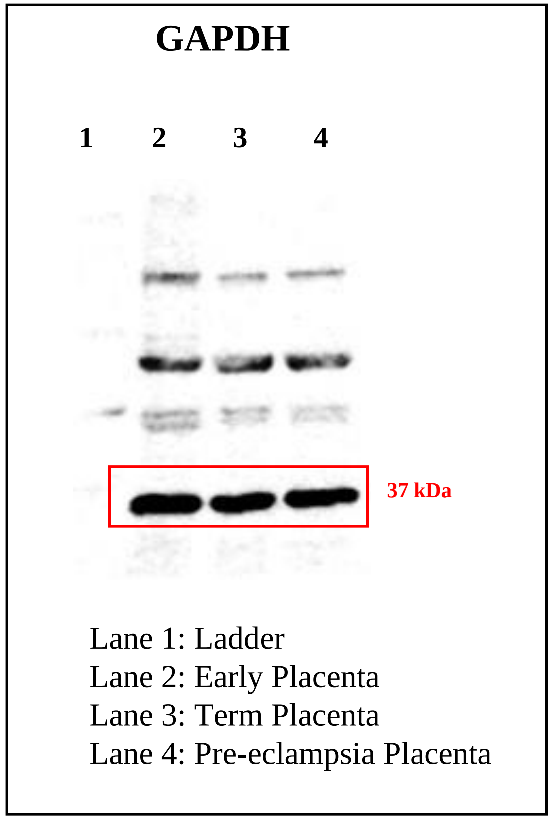
GAPDH
1 2 3 4
37 kDa
Lane 1: Ladder
Lane 2: Early Placenta
Lane 3: Term Placenta
Lane 4: Pre-eclampsia Placenta

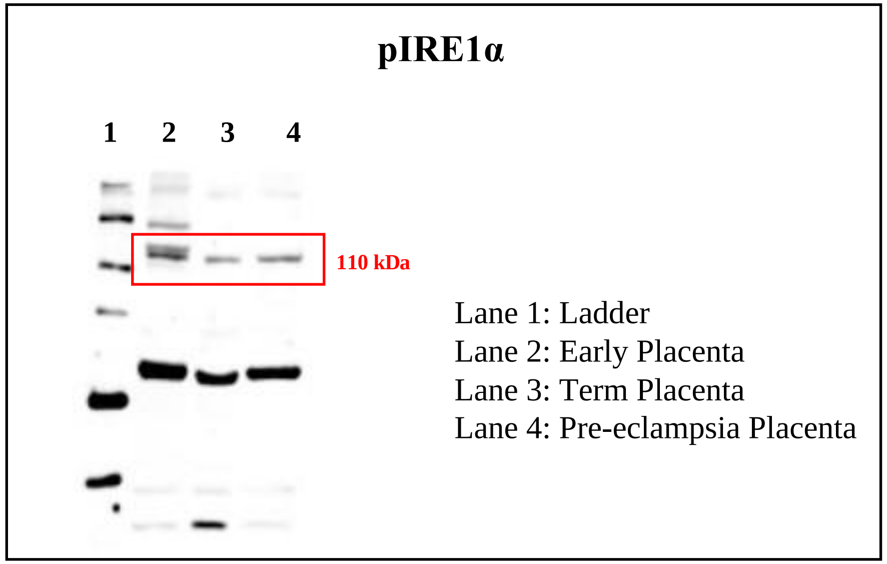
pIRE1α
1 2 3 4
110 kDa
Lane 1: Ladder
Lane 2: Early Placenta
Lane 3: Term Placenta
Lane 4: Pre-eclampsia Placenta

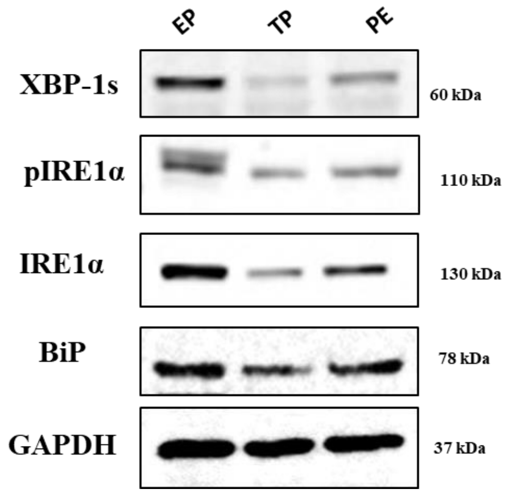
EP
TP
PE
XBP-1s
60 kDa
pIRE1α
110 kDa
IRE1α
130 kDa
BiP
78 kDa
GAPDH
37 kDa

Figure 4a.

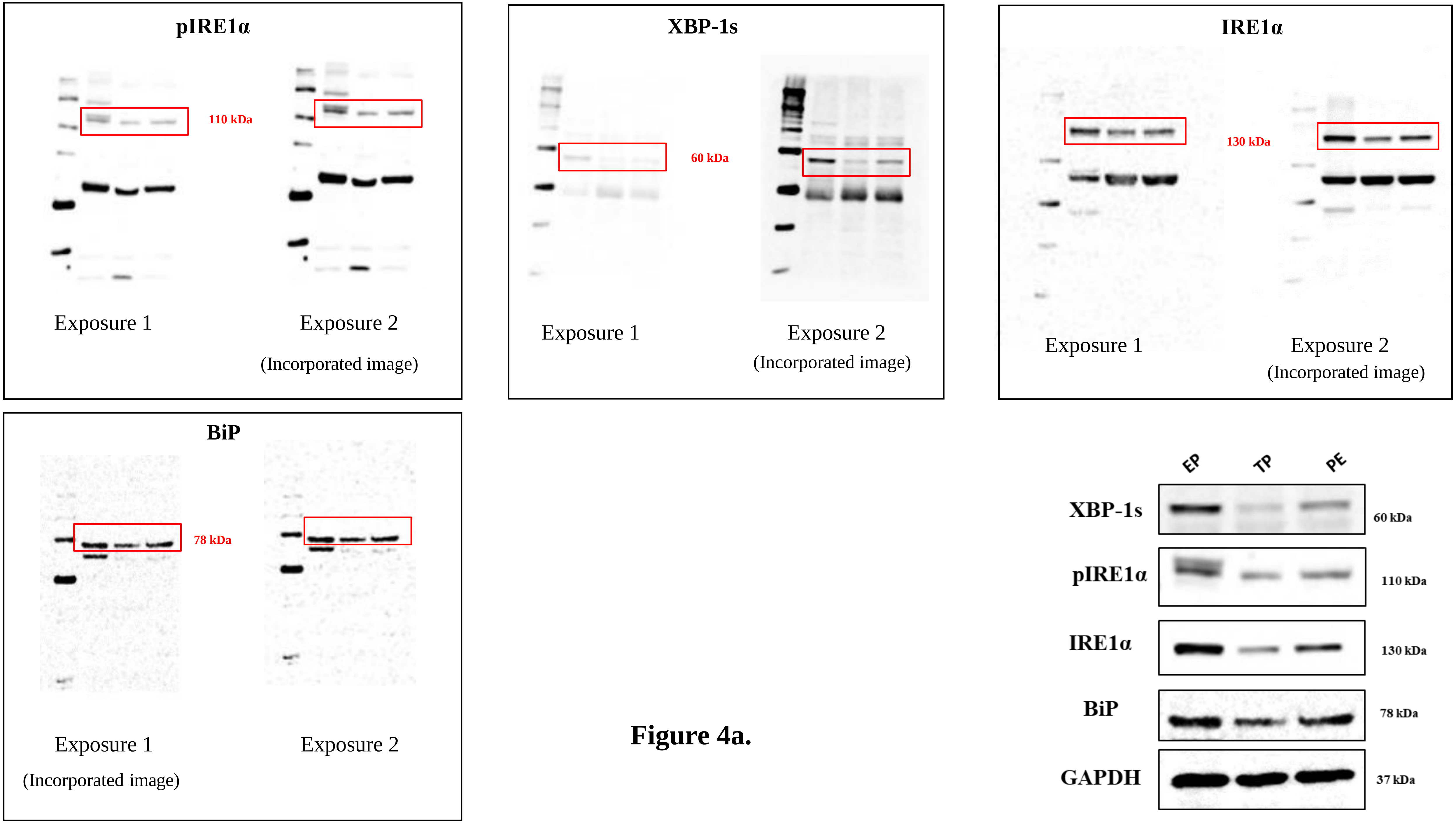
pIRE1α
110 kDa
Exposure 1
Exposure 2
(Incorporated image)
XBP-1s
60 kDa
Exposure 1
Exposure 2
(Incorporated image)
IRE1α
130 kDa
Exposure 1
Exposure 2
(Incorporated image)
BiP
78 kDa
Exposure 1
(Incorporated image)
Exposure 2
EP
TP
PE
XBP-1s
60 kDa
pIRE1α
110 kDa
IRE1α
130 kDa
BiP
78 kDa
GAPDH
37 kDa

Figure 4a.

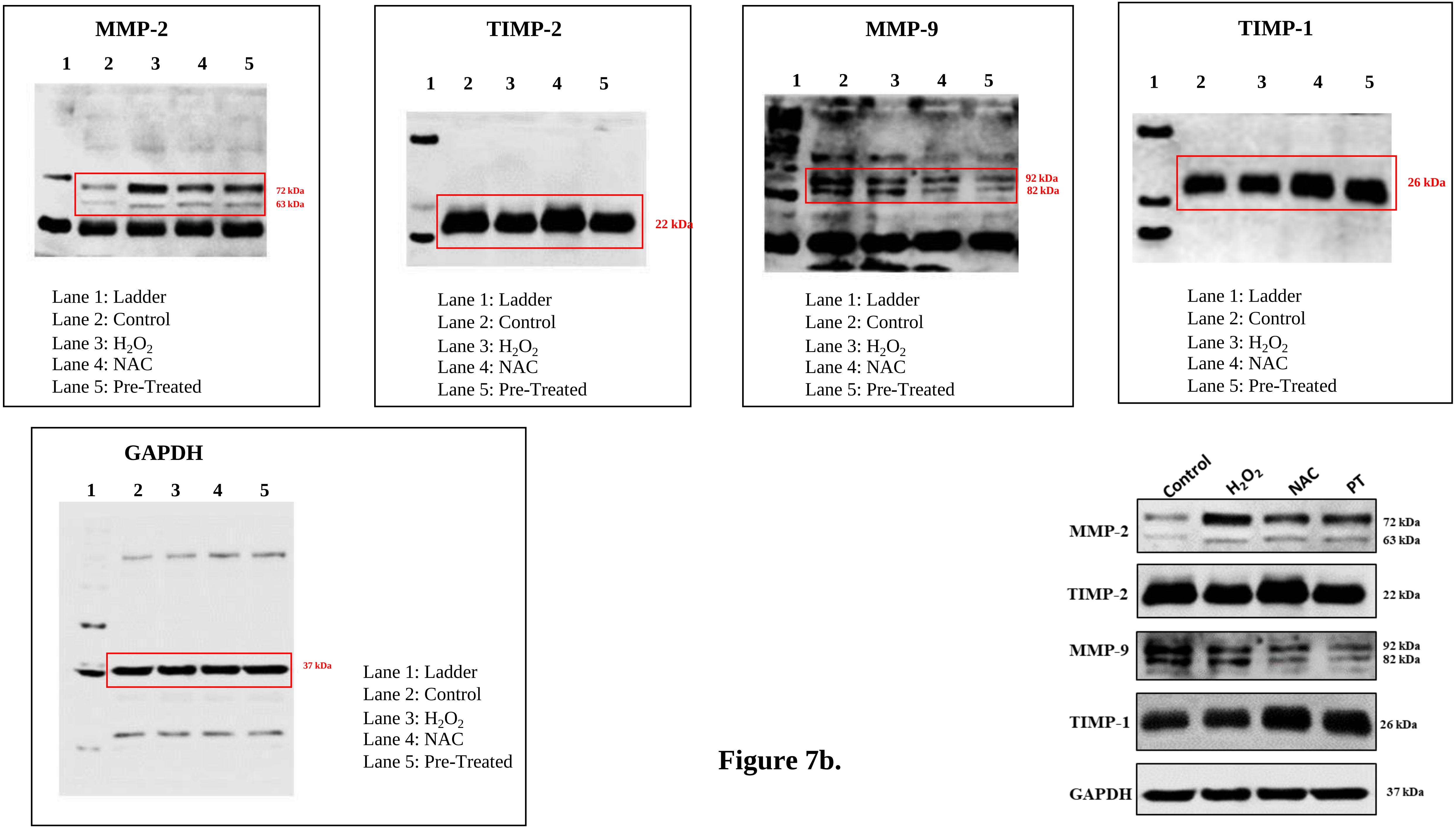
MMP-2
1 2 3 4 5
72 kDa
63 kDa
Lane 1: Ladder
Lane 2: Control
Lane 3: H_2O_2
Lane 4: NAC
Lane 5: Pre-Treated
TIMP-2
1 2 3 4 5
22 kDa
Lane 1: Ladder
Lane 2: Control
Lane 3: H_2O_2
Lane 4: NAC
Lane 5: Pre-Treated
MMP-9
1 2 3 4 5
92 kDa
82 kDa
Lane 1: Ladder
Lane 2: Control
Lane 3: H_2O_2
Lane 4: NAC
Lane 5: Pre-Treated
TIMP-1
1 2 3 4 5
26 kDa
Lane 1: Ladder
Lane 2: Control
Lane 3: H_2O_2
Lane 4: NAC
Lane 5: Pre-Treated
GAPDH
1 2 3 4 5
37 kDa
Lane 1: Ladder
Lane 2: Control
Lane 3: H_2O_2
Lane 4: NAC
Lane 5: Pre-Treated
Control
H_2O_2
NAC
PT
MMP-2
72 kDa
63 kDa
TIMP-2
22 kDa
MMP-9
92 kDa
82 kDa
TIMP-1
26 kDa
GAPDH
37 kDa

Figure 7b.

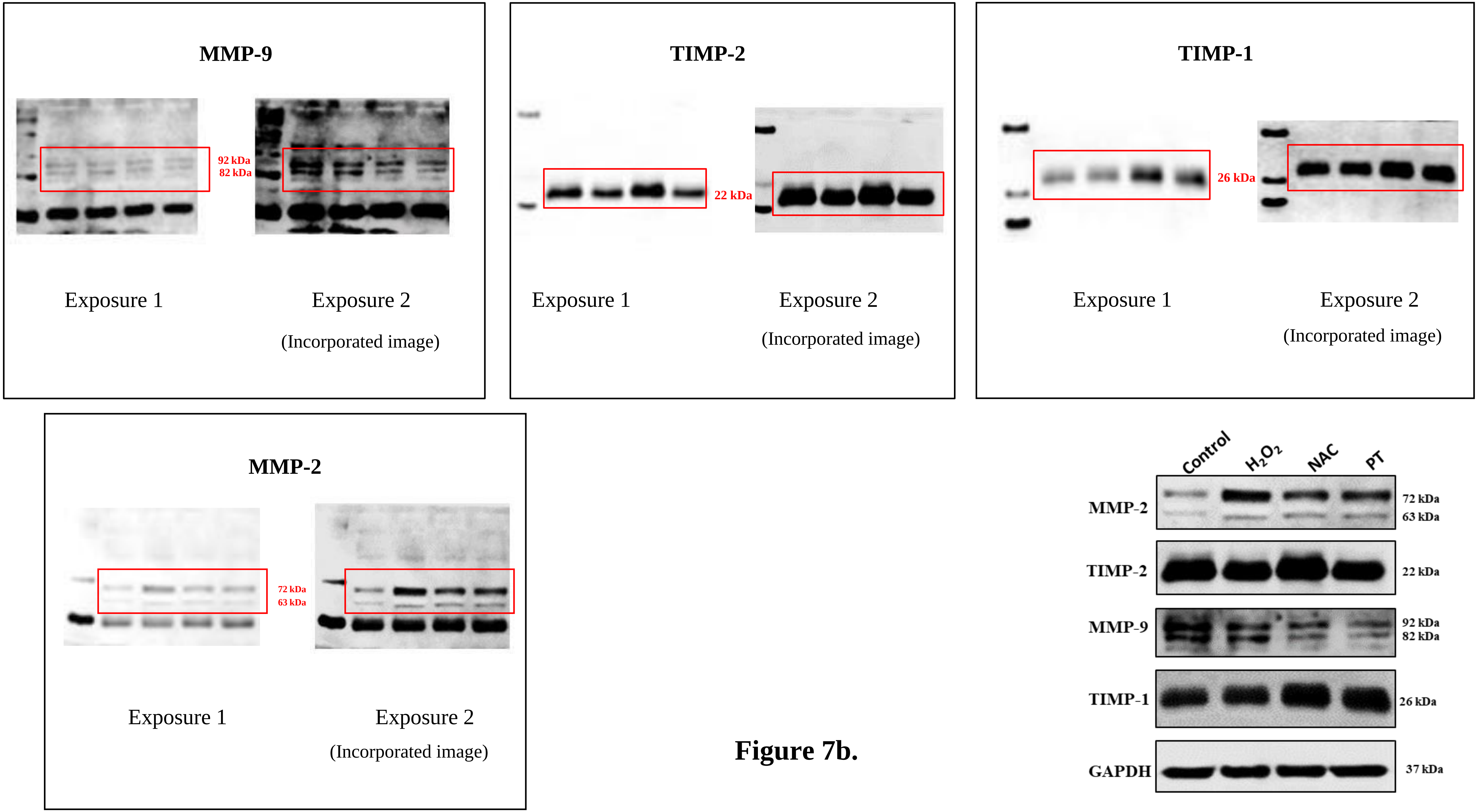

MMP-9
92 kDa
82 kDa
Exposure 1
Exposure 2
(Incorporated image)
TIMP-2
22 kDa
Exposure 1
Exposure 2
(Incorporated image)
TIMP-1
26 kDa
Exposure 1
Exposure 2
(Incorporated image)
MMP-2
72 kDa
63 kDa
Exposure 1
Exposure 2
(Incorporated image)
Control
H2O2
NAC
PT
MMP-2
72 kDa
63 kDa
TIMP-2
22 kDa
MMP-9
92 kDa
82 kDa
TIMP-1
26 kDa
GAPDH
37 kDa

Figure 7b.

Figure. 7n

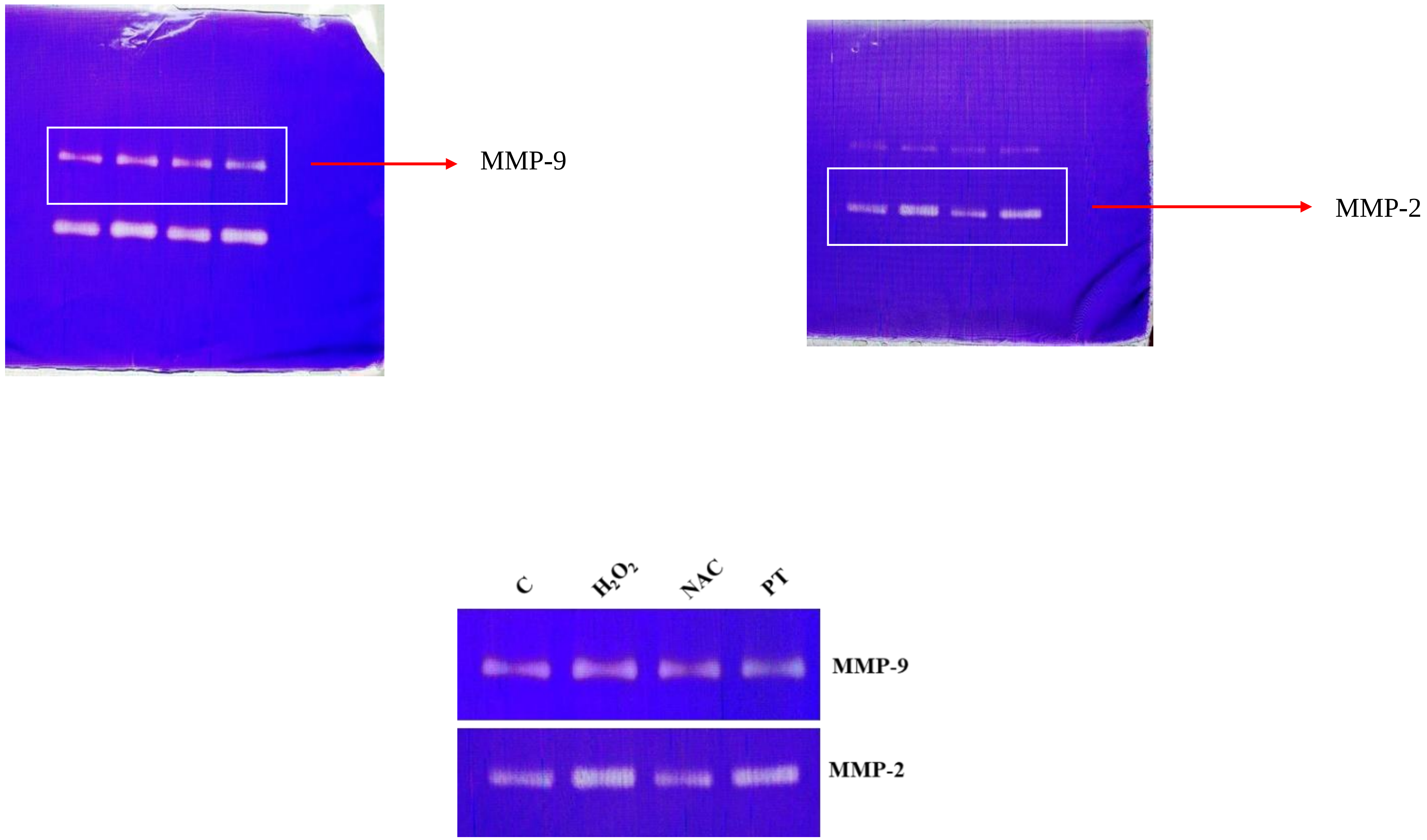

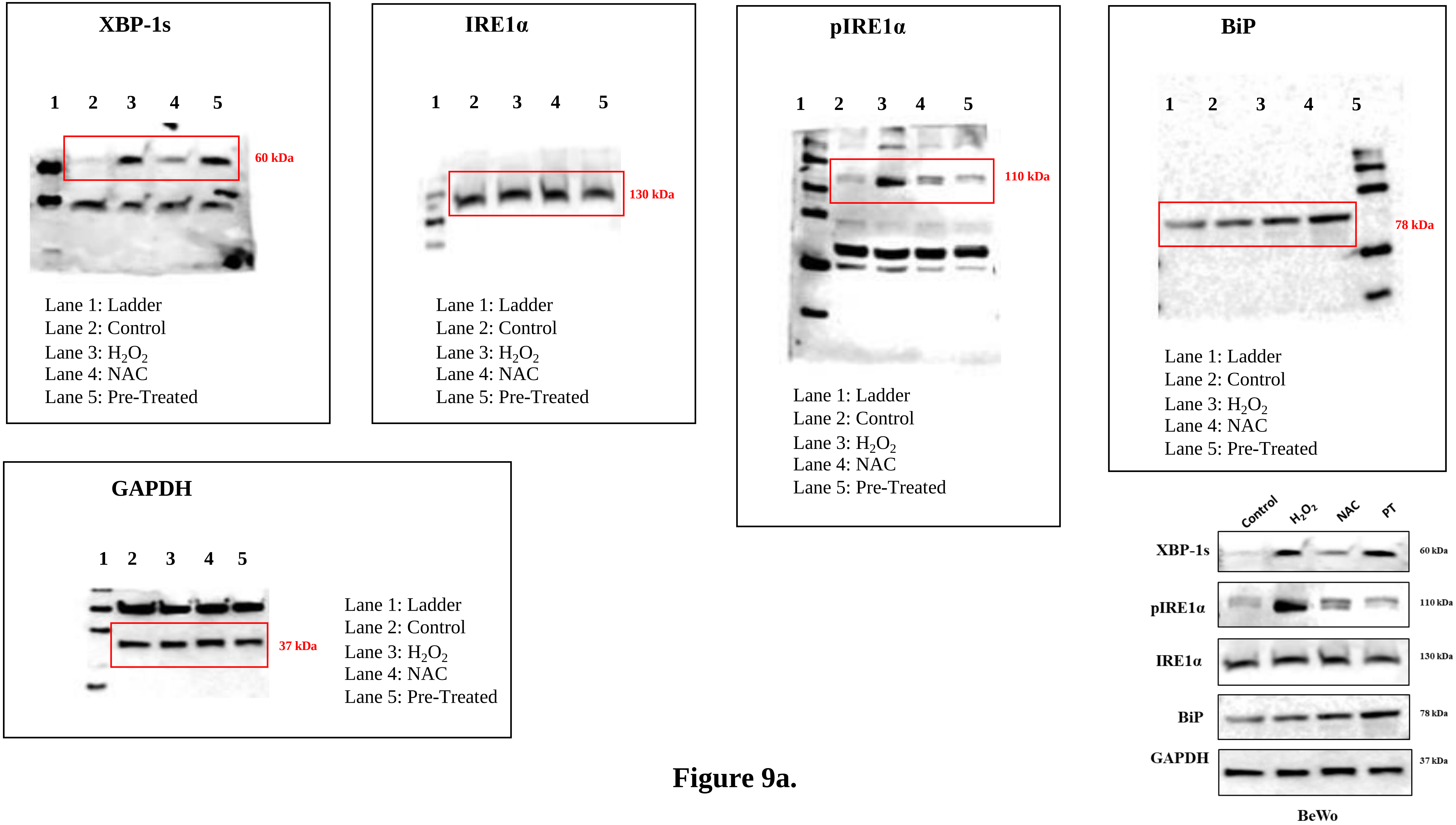
XBP-1s
1 2 3 4 5
60 kDa
Lane 1: Ladder
Lane 2: Control
Lane 3: H_2O_2
Lane 4: NAC
Lane 5: Pre-Treated
IRE1α
1 2 3 4 5
130 kDa
Lane 1: Ladder
Lane 2: Control
Lane 3: H_2O_2
Lane 4: NAC
Lane 5: Pre-Treated
pIRE1α
1 2 3 4 5
110 kDa
Lane 1: Ladder
Lane 2: Control
Lane 3: H_2O_2
Lane 4: NAC
Lane 5: Pre-Treated
BiP
1 2 3 4 5
78 kDa
Lane 1: Ladder
Lane 2: Control
Lane 3: H_2O_2
Lane 4: NAC
Lane 5: Pre-Treated
GAPDH
1 2 3 4 5
37 kDa
Lane 1: Ladder
Lane 2: Control
Lane 3: H_2O_2
Lane 4: NAC
Lane 5: Pre-Treated
Control
H_2O_2
NAC
PT
XBP-1s
60 kDa
pIRE1α
110 kDa
IRE1α
130 kDa
BiP
78 kDa
GAPDH
37 kDa
BeWo

Figure 9a.

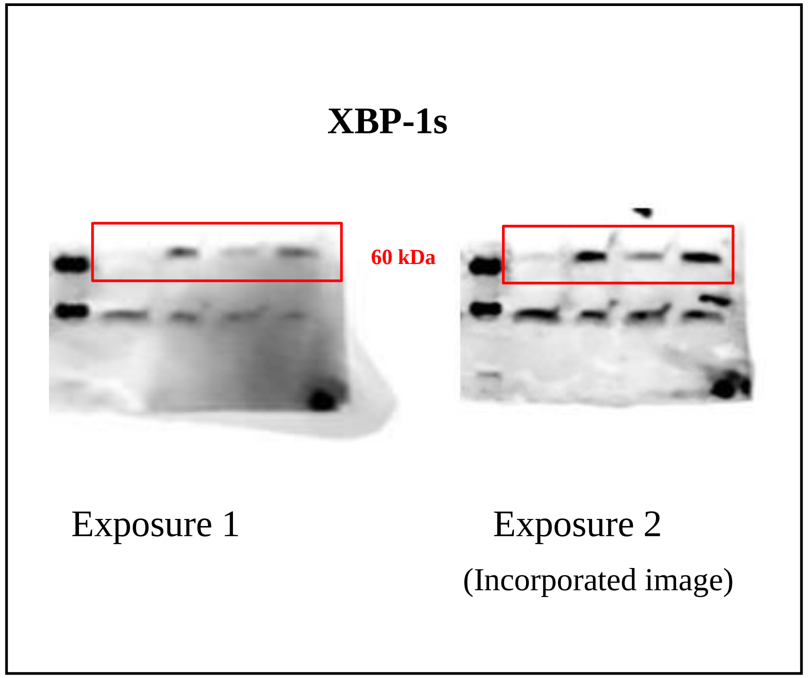
XBP-1s
60 kDa
Exposure 1
Exposure 2
(Incorporated image)

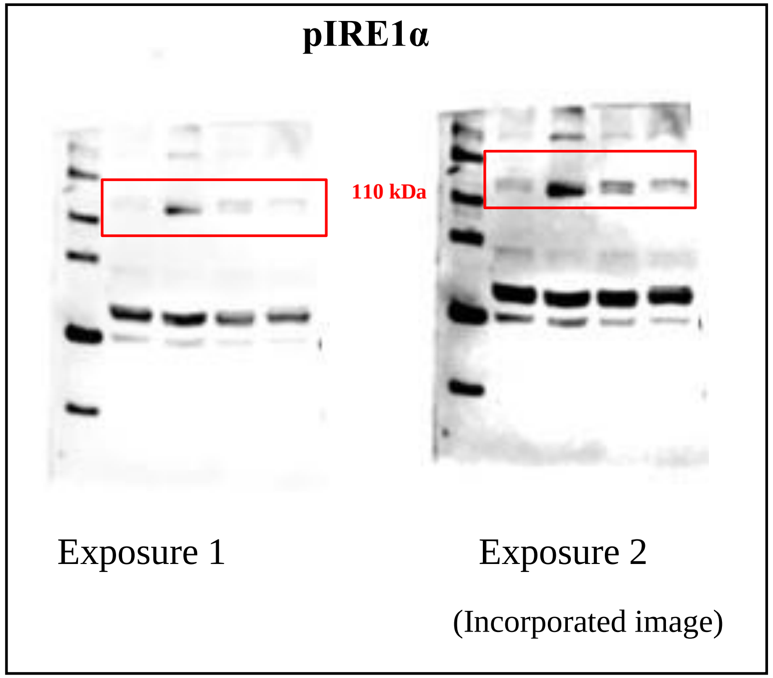
pIRE1α
110 kDa
Exposure 1
Exposure 2
(Incorporated image)

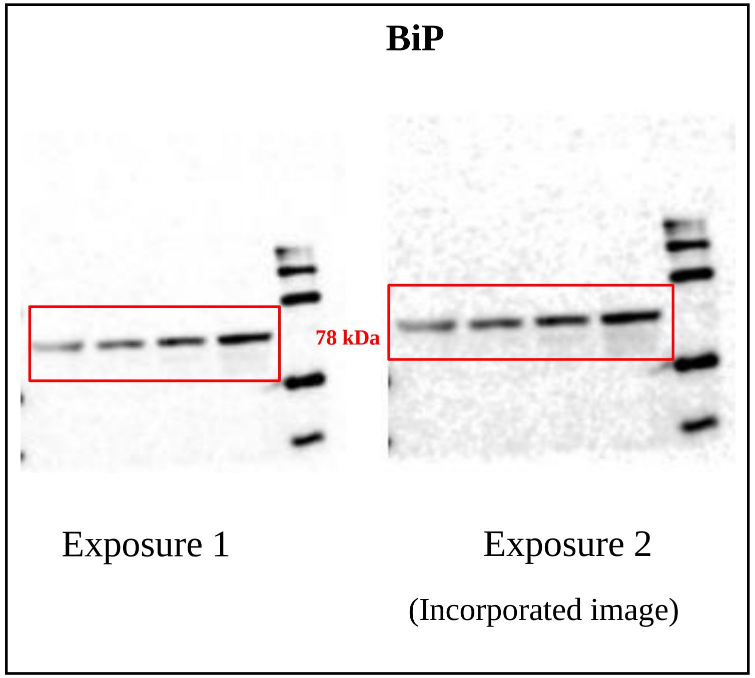
BiP
78 kDa
Exposure 1
Exposure 2
(Incorporated image)

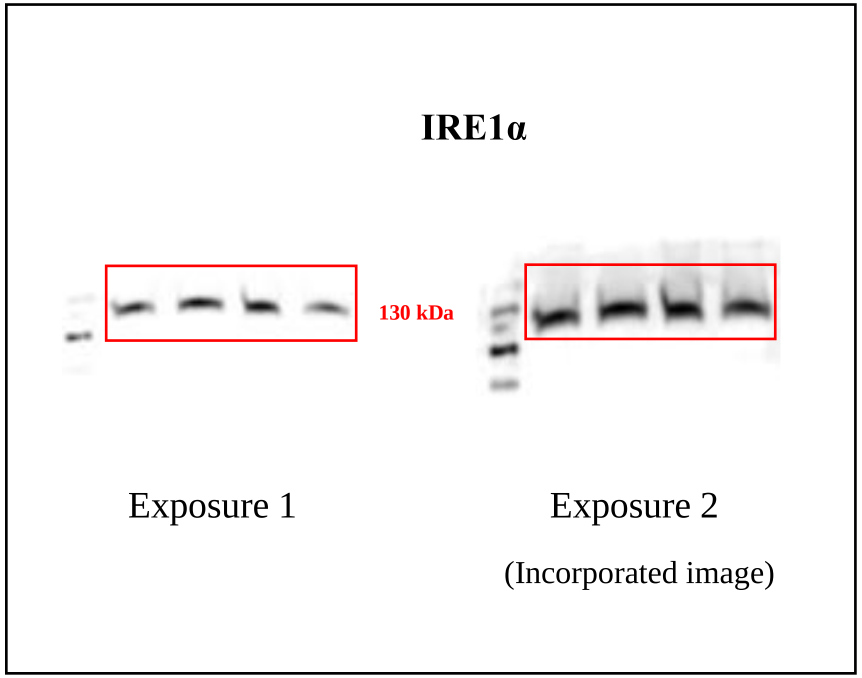
IRE1α
130 kDa
Exposure 1
Exposure 2
(Incorporated image)

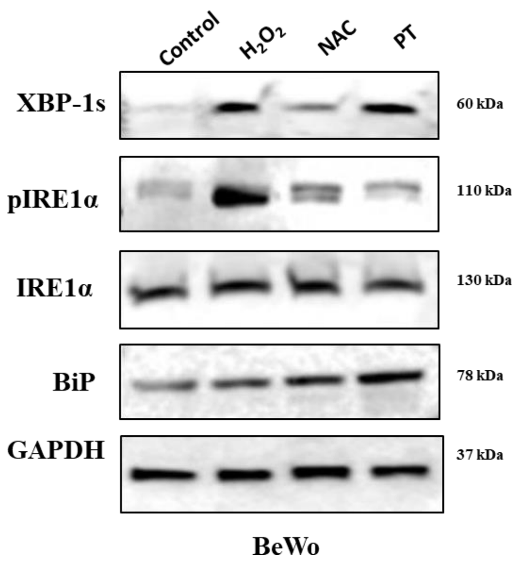
Control
H2O2
NAC
PT
XBP-1s
60 kDa
pIRE1α
110 kDa
IRE1α
130 kDa
BiP
78 kDa
GAPDH
37 kDa
BeWo

Figure 9a.

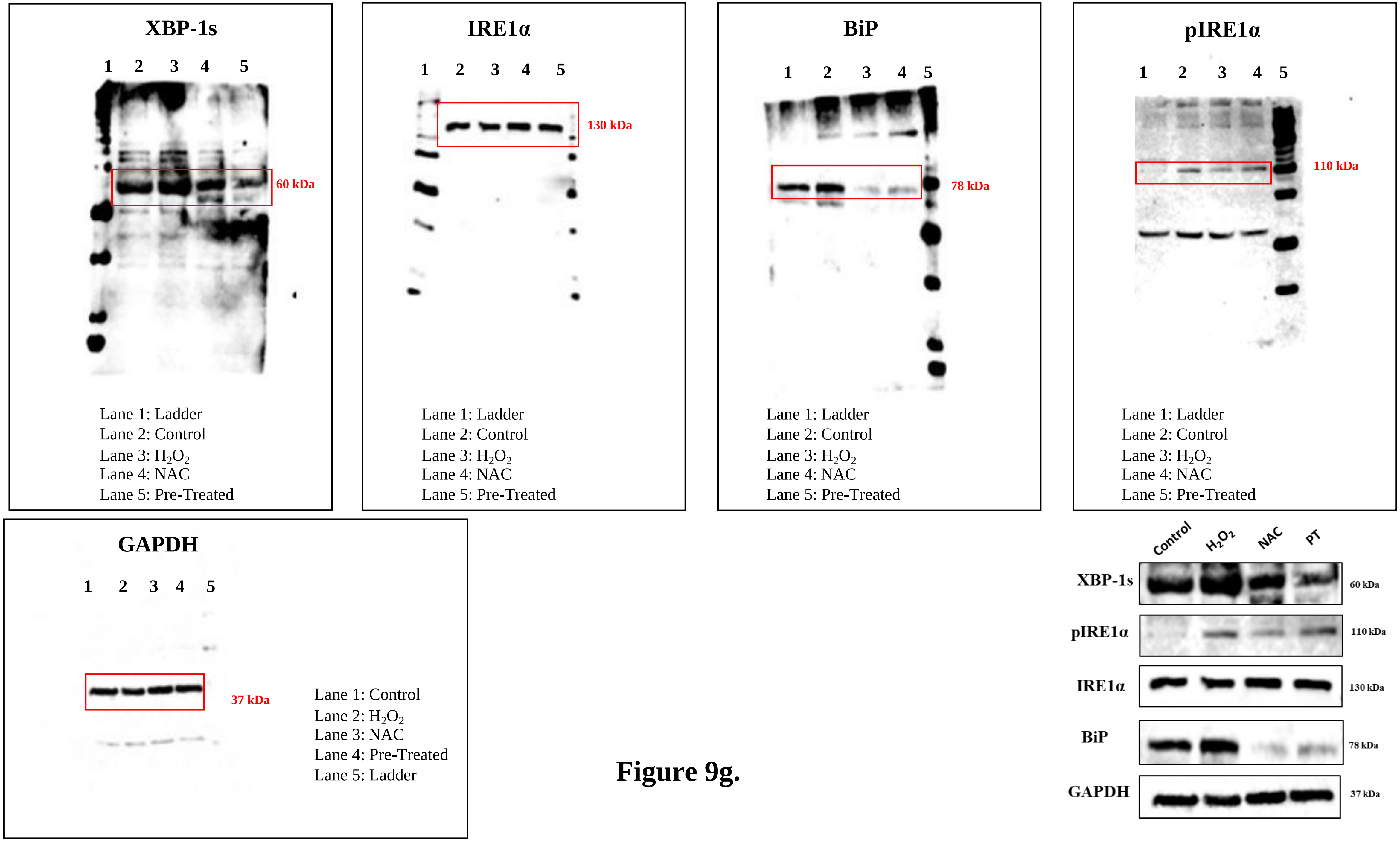
XBP-1s
1 2 3 4 5
60 kDa
Lane 1: Ladder
Lane 2: Control
Lane 3: H_2O_2
Lane 4: NAC
Lane 5: Pre-Treated
IRE1α
1 2 3 4 5
130 kDa
Lane 1: Ladder
Lane 2: Control
Lane 3: H_2O_2
Lane 4: NAC
Lane 5: Pre-Treated
BiP
1 2 3 4 5
78 kDa
Lane 1: Ladder
Lane 2: Control
Lane 3: H_2O_2
Lane 4: NAC
Lane 5: Pre-Treated
pIRE1α
1 2 3 4 5
110 kDa
Lane 1: Ladder
Lane 2: Control
Lane 3: H_2O_2
Lane 4: NAC
Lane 5: Pre-Treated
GAPDH
1 2 3 4 5
37 kDa
Lane 1: Control
Lane 2: H_2O_2
Lane 3: NAC
Lane 4: Pre-Treated
Lane 5: Ladder
Control
H_2O_2
NAC
PT
XBP-1s
60 kDa
pIRE1α
110 kDa
IRE1α
130 kDa
BiP
78 kDa
GAPDH
37 kDa

Figure 9g.

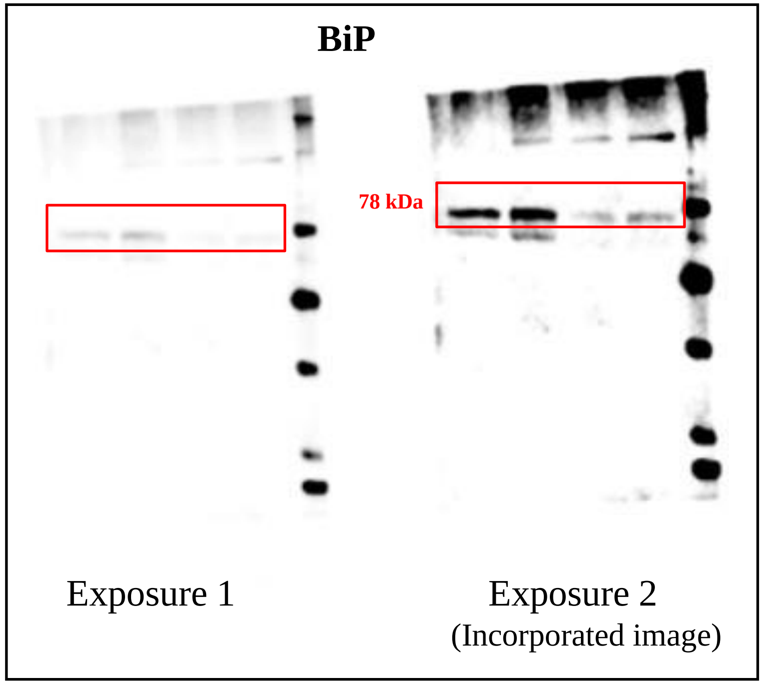
BiP
78 kDa
Exposure 1
Exposure 2
(Incorporated image)

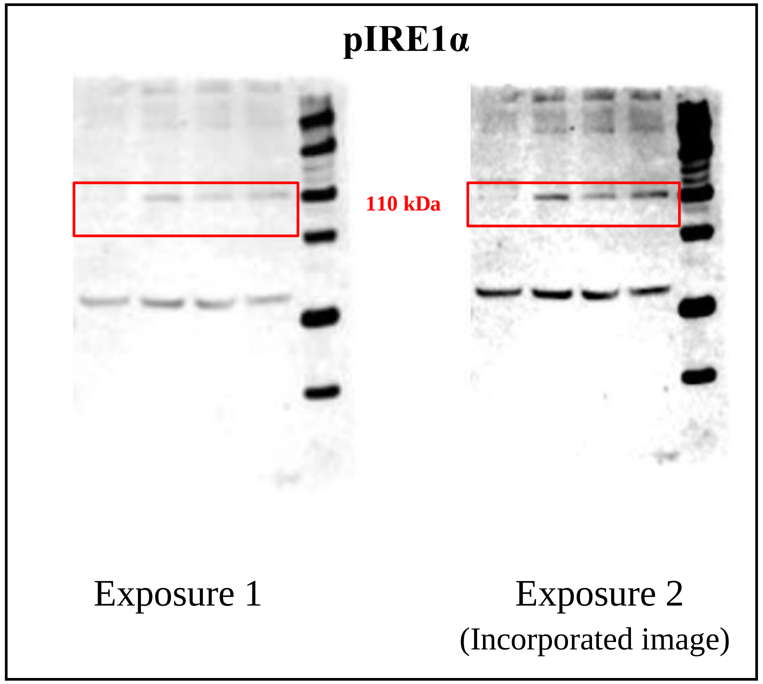
pIRE1α
110 kDa
Exposure 1
Exposure 2
(Incorporated image)

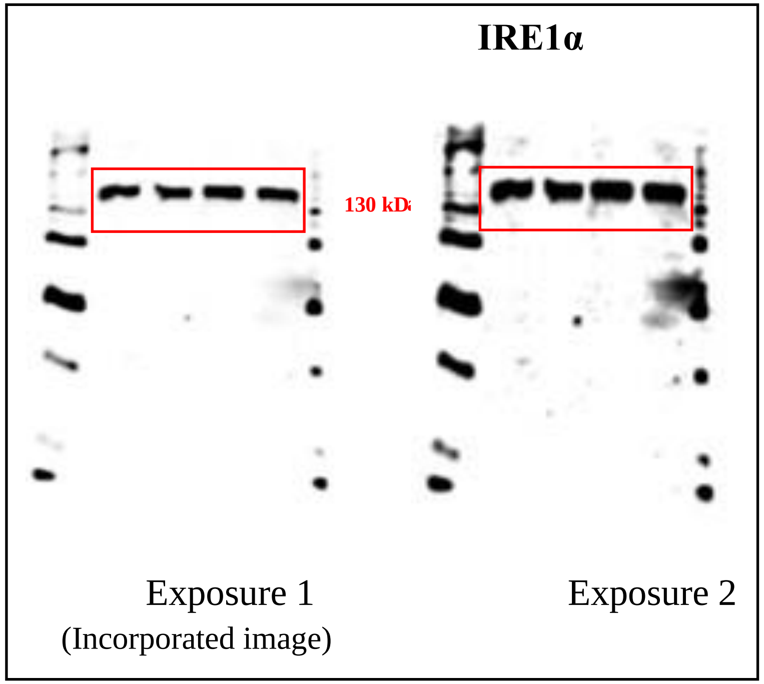
IRE1α
130 kDa
Exposure 1
(Incorporated image)
Exposure 2

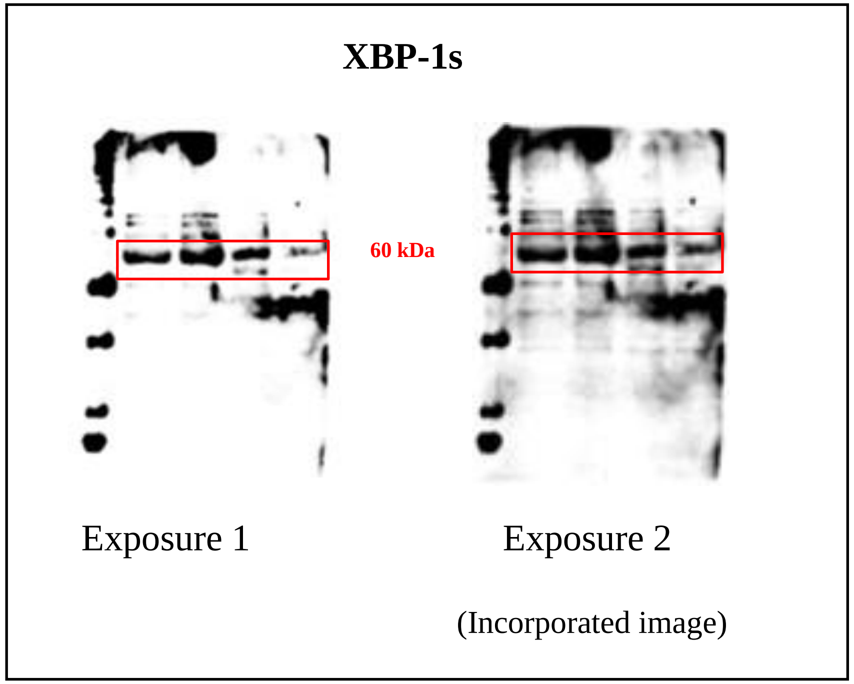
XBP-1s
60 kDa
Exposure 1
Exposure 2
(Incorporated image)

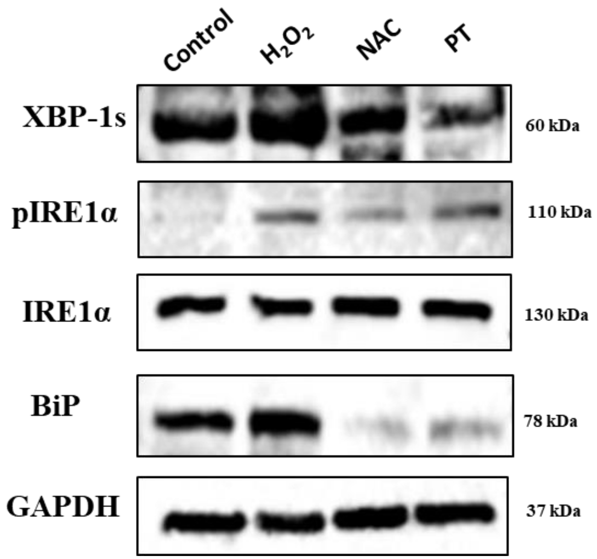
Control
H2O2
NAC
PT
XBP-1s
60 kDa
pIRE1α
110 kDa
IRE1α
130 kDa
BiP
78 kDa
GAPDH
37 kDa

Figure 9g.

GAPDH for pIRE1α

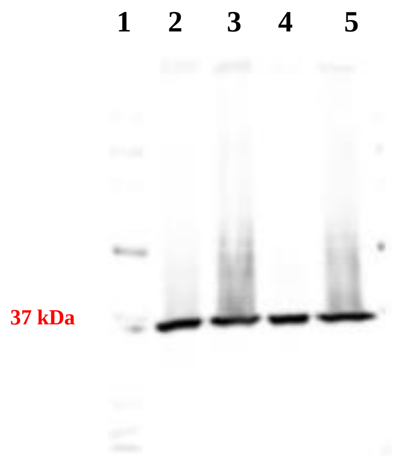

Lane 1: Ladder
Lane 2: Control
Lane 3: H_2O_2
Lane 4: NAC
Lane 5: Pre-Treated

BeWo

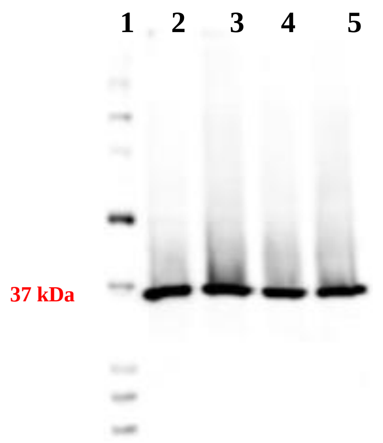

Lane 1: Ladder
Lane 2: Control
Lane 3: H_2O_2
Lane 4: NAC
Lane 5: Pre-Treated

HTR-8/SV neo

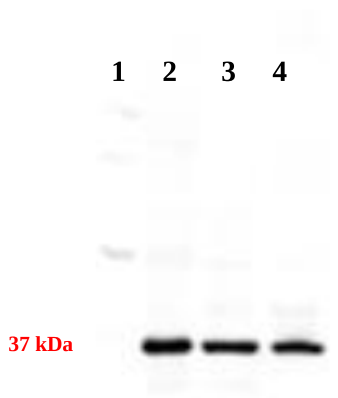

Lane 1: Ladder
Lane 2: Early Placenta
Lane 3: Term Placenta
Lane 4: Pre-eclampsia Placenta

Tissues
